# Exomes in Paediatrics: Co‐Design and Implementation of Interventions to Support Paediatricians to Provide Genomic Care

**DOI:** 10.1111/jpc.70237

**Published:** 2025-11-20

**Authors:** Belinda Dawson‐McClaren, Melissa Martyn, Emma Weisz, Rigan Tytherleigh, Justine Elliott, Heather Renton, Natasha J. Brown, Michael Fahey, Clara Gaff

**Affiliations:** ^1^ Murdoch Children's Research Institute Melbourne Victoria Australia; ^2^ Melbourne Genomics Health Alliance, Walter and Eliza Hall Institute Melbourne Victoria Australia; ^3^ University of Melbourne Melbourne Victoria Australia; ^4^ Monash Children's Hospital Melbourne Victoria Australia; ^5^ Victorian Clinical Genetics Service Melbourne Victoria Australia; ^6^ Syndromes Without A Name (SWAN) Melbourne Victoria Australia

**Keywords:** co‐design, evaluation, genomics, intervention, paediatricians

## Abstract

**Background:**

Despite the potential benefits of genomic testing for children with unique body features and/or developmental differences, these tests are underutilised by paediatricians.

**Aim:**

To co‐design, implement and evaluate interventions to support paediatricians in overcoming barriers to requesting funded genomic tests.

**Methods:**

A mixed‐methods approach was taken. Co‐design workshops with paediatricians, parents and genetic health professionals informed the development of multi‐modal interventions. Evaluation, guided by the RE‐AIM framework: data were surveys, website analytics, test request audits and interviews.

**Results:**

The multi‐modal intervention was a website, genetic expert consultation service and teaching clinic. Implementation of the intervention was supported by awareness‐raising activities. Evaluation showed the website was accessed by 222 paediatricians in the first 12 months. Survey responses (T1, 1‐month post‐access: 11/218, 5%; T2, 3‐months post‐access: 14/218, 6%) indicated that confidence in knowledge‐based areas increased and remained elevated 3 months after website access; skill‐based confidence was also elevated but not sustained. The genetic expert consultation service, though accessed less than anticipated, provided valued support. The teaching clinic in metropolitan Melbourne offered experiential learning opportunities aimed at skill development. The clinic had 62 appointments with 39 patients. Thirteen paediatricians attended the clinic for experiential learning and 9 set learning goals. During the intervention period, requests for funded genomic tests by paediatricians increased by 227% compared to pre‐intervention. Qualitative data (interviews with 12 paediatricians and 4 genetic experts) revealed paediatricians gained practical skills and confidence to assess child eligibility, discuss testing with parents and complete test requests.

**Conclusions:**

The interventions helped increase paediatrician confidence and skills, highlighting the importance of combining knowledge transfer with experiential learning to support comprehensive practice change. These results offer a foundation for ongoing efforts to support wider, more equitable use of genomic tests. Long‐term evaluation would further assess the impact on paediatrician practice. The study demonstrates the value of co‐designed, multi‐modal interventions in addressing complex practice changes in healthcare.

**Summary**

- What's already known on this topic
  ○ Genomic testing can significantly impact treatment and management for children with childhood syndromes, with diagnostic yields ranging from 10% 60%.
  ○ In Australia, paediatricians can directly request genomic testing (in consultation with a clinical geneticist), but ordering rates remain lower than expected, suggesting barriers to practice.
  ○ To develop confidence and skills in a new area of practice, providing educational materials will not fully address these needs; experiential learning is critical.
- What this paper adds
  ○ A co‐designed, multi‐modal intervention approach (website, genetic expert consultation service and teaching clinic) can increase paediatricians' confidence and skills to order funded genomic tests.
  ○ To implement interventions, active awareness‐raising methods, such as clinic and department meetings with genetic experts present, are more effective than passive methods in promoting awareness of interventions.
  ○ Insights into the implementation and evaluation of interventions supporting paediatricians in genomic testing, offering a foundation for ongoing efforts to promote wider, more equitable use of genomic tests for children with developmental delay ± unique body features.

## 1 Introduction

Genomic testing can substantially impact treatment and management for children with childhood syndromes, with diagnostic yields ranging from 10% to 60% [1, 2, 3]. However, long wait times for clinical genetics appointments hinder timely access to these tests. In Australia, paediatricians can directly request funded genomic tests, but ordering rates remain considerably lower than expected, suggesting barriers to practice [4]. Our previous study identified individual (limited knowledge and skills, perception of scope of role, belief about value of testing) and service‐level barriers (pressures on appointment length and frequency, absence of interprofessional connections) experienced by paediatricians requesting funded genomic tests in Australia [5]. These are consistent with barriers described above across broad applications of genomic medicine. In those interviews, paediatricians suggested strategies to support their practice should be focused on: awareness raising, education and knowledge building, practical support and resources that can be shared with families.

We describe here:

1. Our process of co‐design to develop interventions addressing those topics (File S1).
2. The resultant interventions.
3. A mixed methods evaluation of the implementation of the interventions.

## 2 Method

### 2.1 Co‐Design of Interventions

Full details of the co‐design approach, participant recruitment, activities and output are described in File S1. Evidence‐based co‐design was the approach used and involves co‐designers engaging in participatory activities including journey mapping, storytelling, identifying touch points, prototyping interventions and identifying barriers and enablers [6]. Specifically, we conducted online workshops (2 h in duration) with paediatricians, parents/consumer representatives (referred to as ‘parents’ herein) and genetic health professionals. Using convenience sampling participants were recruited because they had previously participated in an interview study (paediatricians), responded to an expression of interest advertising the study (parents), or were known to work at a genetic service in a role related to exome testing for childhood syndromes (genetic health professionals). These were facilitated by co‐design experts from the University of Melbourne Methods and Implementation Support for Clinical and Health Research (MISCH). The different stakeholder groups attended separate workshops. Co‐design Cycle 1 workshops gathered information around needs and potential interventions. Co‐design Cycle 2 discussed design principles for specific interventions and commenced direct development of interventions in which paediatricians and parents reviewed draft content for information delivered in a website format; genetic health professionals participated in in‐person meetings (2 h in duration) to explore a phone/email based genetic expert consultation service for paediatricians.

### 2.2 The Intervention

A multi‐modal intervention was developed:

1. A website with content informed by co‐design, and authored by study paediatrician EW, providing information and existing resources for requesting funded genomic testing (www.paediatricgenomics.org.au). File S2 provides examples of website content.
2. A secondary consultation service staffed by genetic experts (genetic counsellors + scientists/liaison staff), and accessible via phone, email or webform.
3. A joint paediatrics–genetics teaching clinic established at a tertiary public hospital.

The website featured a public landing page and for full access required users to provide an email address, name and practice location via a pop‐up. Instant access was granted and these data were used to verify users as paediatricians, and to send evaluation surveys. The secondary consultation service leveraged an existing service at the clinical genetic laboratory service with additional staffing (a genetic counsellor) provided by and for the study. The teaching clinic (April 2024 to February 2025), led by a paediatrician with genomics expertise under the governance of a clinical genetics service, offered experiential learning for referring paediatricians. Paediatrician participants received pre‐clinic education modules and individualised learning experiences, driven by goal setting, during consultations.

Further details about the teaching clinic are provided in a companion letter to the editor (to be submitted). Briefly, the clinic was located at a metropolitan Melbourne hospital site that has a genetics and general paediatric unit, and author E.W. has established relationships with both departments. The referral list of the genetic service was reviewed and families of children eligible for Medicare‐funded exome testing were invited to attend the paediatric exome teaching clinic. Their referring paediatrician was also invited to attend; however it was not a requirement for the appointment to proceed, and a comprehensive letter was provided by EW to the referring paediatrician after the appointment. The clinic was held weekly, 3.5 h in duration and involves a co‐consultation between EW and an experienced genetic counsellor. Remote support by a clinical geneticist occurs with weekly case discussions.

### 2.3 Implementation of Interventions

Interventions were promoted through:

1. Presentations (E.W. and J.E.) at professional development events, clinic meetings and department sessions across Victoria.
2. Indirect methods including newsletter items and social media posts.
3. Targeted LinkedIn advertisements for professionals identified as paediatricians or working in paediatric care in Victoria.

These promotional activities aimed to raise awareness about the interventions and encourage their use.

### 2.4 Mixed‐Methods Evaluation of Intervention

The framework elements, with a specific research question defined for each are:

- *Reach*: At the individual level, did the interventions reach our target population of general paediatricians?
- *Efficacy/Effectiveness*: What was the change (positive, negative, neutral) in paediatrician confidence and familiarity following intervention access?
- *Adoption*: Did paediatricians, and across what settings, use the intervention(s)?
- *Implementation*: Were the interventions used as intended? For example, what was used, how was it used and what wasn't used?
- *Maintenance*: Has there been a change in confidence and practice (test requests) over time?

File S3 provides a description of the RE‐AIM framework and modifications for use in this study. The framework consists of five key outcomes: Reach, Effectiveness, Adoption, Implementation and Maintenance, and is most commonly used as an evaluative framework for the implementation of evidence‐based interventions. Use of the framework does not require all outcomes to be measured, or fully measured, and mixed methods (e.g., surveys and interviews) are appropriate to investigate outcomes where possible, and/or expectations and perceptions of the outcomes. Our study has used the RE‐AIM framework in this way to evaluate the implementation of the intervention, with the available data sources over the available study period. The RE‐AIM framework ensured that the evaluation was comprehensive and pragmatic by capturing individual and system‐level factors to describe the impact of the interventions on paediatrician behaviour [7]. A mixed‐methods approach collected data with minimal user disruption. The primary outcome was paediatrician‐reported confidence and practice changes in the short term. Quantitative data were captured about the awareness‐raising activities, use of the website (Google Analytics), surveys for paediatricians to complete 1 and 3 months after website access and, an audit of test requests coded to the Medicare item numbers for childhood syndromes (laboratory data of paediatrician‐ordered tests, 12 June 2022 to 3 March 2025) made before, during and after intervention implementation. The percentage change of test requests was calculated for each period. Survey measures and interview topics addressed changes in paediatricians' confidence, skills, behaviour/practice, use of interventions, and encountered barriers and enablers. Qualitative data were collected through interviews with paediatricians and genetic experts, practice reflection notes of the study genetic experts and learning goals of referring paediatricians. All genetic experts responsible for the consultation service were invited for evaluation interviews. Maximum variation sampling was used to invite paediatricians who had engaged with the intervention and provided contact information. Variation was sought based on the intervention component they engaged with (website, consultation service and teaching clinic).

## 3 Results

### 3.1 Co‐Design

Co‐design participants were invited to all events relevant to their stakeholder group; however they were not all available to attend all activities. In total there was co‐design participation in one or more online activities by 21 participants: 8 parents, 5 paediatricians and 8 genetic health professionals.

Cycle 1, 2‐h workshops were attended by three paediatricians, four genetic health professionals and six parents. Cycle 2 was attended by seven individuals: three paediatricians (one from Cycle 1), six genetic health professionals (two from Cycle 1) and four parents (two from Cycle 1). A comparison table of the co‐design proposed strategies and the resultant multi‐modal intervention is shown in File S1.

### 3.2 Evaluation

Twelve paediatricians, who engaged with components of the intervention and four genetic experts providing the consultation intervention, were interviewed from July 2024 to March 2025. The interview guides are provided in File S4. Representative quotes are used throughout the article, with further examples provided in File S5.

### 3.3 Reach

**FIGURE 1:**
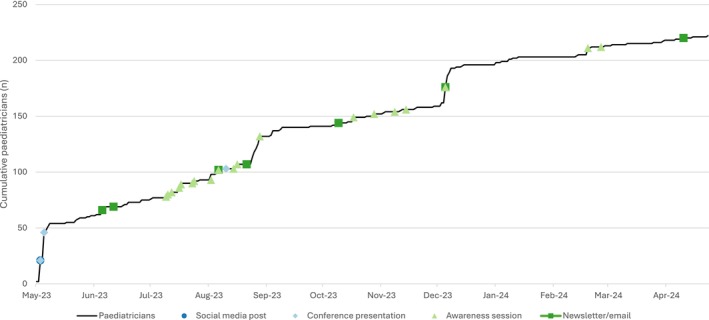
Timeline of awareness‐raising activities and corresponding access of website by paediatricians.

Over 12 months from May 2023, 2 conferences and 15 small group events reached approximately 400 individuals across Victoria (Figure 1). In total, 222 verified paediatricians accessed the step‐by‐step guide: 127 (57.2%) from Victoria (98 metropolitan, 29 regional) and 95 (42.8%) from interstate.

The website was presented in detail at the awareness raising events. Prior to the presentation, attending paediatricians completed a baseline survey. Table 1 shows the baseline survey results related to current and anticipated future practice.

**TABLE 1:** Baseline survey data related to current and anticipated future practice.

Characteristic	Responses percent
Previously referred a patient to a clinical genetics service for genomic testing (*n* = 70), *n* (%)	
Yes	56 (80.0%)
No	13 (18.6%)
Unsure	1 (1.4%)
Previously requested a test using Medicare item numbers (*n* = 65), *n* (%)^a^	
Yes	4 (6.2%)
No	45 (69.2%)
Unsure	11 (16.9%)
Not applicable to role	5 (7.7%)
Anticipate ordering Medicare funded tests in the future (*n* = 65), *n* (%)	
Yes	54 (83.1%)
No	2 (3.1%)
Unsure	9 (13.9%)
	*Scale 0–10*
Familiarity with genomic testing (*n* = 71), mean (SD)^b^	3.7 (2.4)
Feel genomic testing will become a normal part of work (*n* = 71), mean (SD)^c^	7.2 (2.3)

^a^ Not asked of all survey respondents, therefore a lower denominator.

^b^ Responses ranged from 0 (still feels very new) to 10 (feels completely familiar).

^c^ Response options ranged from 0 (not at all) to 10 (completely).

### 3.4 Effectiveness

**FIGURE 2:**
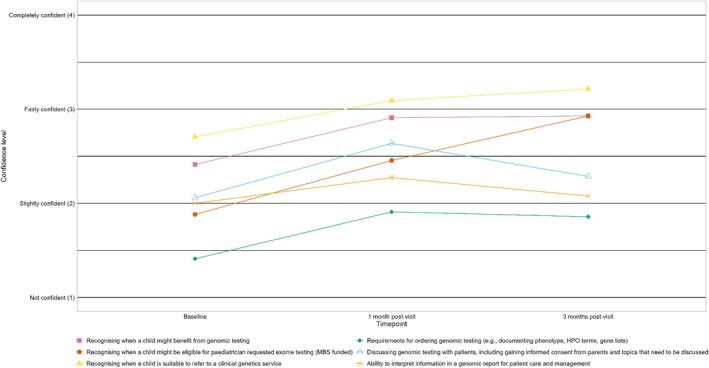
Trends in mean confidence of paediatricians across process steps to request genomic testing.

Surveys assessed paediatricians' confidence in six genomic testing process steps (Figure 2). Response rates were: Baseline: undetermined due to hybrid sessions; T1 (1 month post‐access): 11/218 (5%); T2 (3 months post‐access): 14/218 (6%).

The descriptions provided by paediatricians in their interviews explore these trends further (File S5). For instance, Paediatrician 2 used the website to gain information about their patient's eligibility for funded genomic testing and contacted the genetic expert service for a call‐back to seek support around potential results and how to access support from a genetic counsellor if needed.

I was extremely worried because I was thinking a lot about the possibility of things being discovered in potentially the whole three of them [child and parents] that may not be necessarily relevant to the child's presentation, and ethically, what that might mean… I didn't know that I felt I would be confident enough to be able to counsel them… I looked at the website [to check eligibility]… And then also I [contacted the genetic expert service and] then my anxiety level dropped by like 80% after I had that phone call. I just suddenly realised like ‘ohh great, if we get something back on this, I'm not by myself’… the confidence just went right up. (Paediatrician 2)

### 3.5 Adoption

In the first 12 months, the website had 5257 page views and captured 264 unique email addresses of which 222 (84%) were manually verified as belonging to a paediatrician. Therefore most views and downloads to the website were from paediatricians.

The genetic expert consultation service had nine contacts directly resulting from access to the website or attendance at an awareness‐raising activity from July 2023 to January 2024. Despite the low volume of access, these were high‐quality interactions to discuss specific patients and/or challenges with the ordering process. An example is provided in Box 1.

BOX 1 **Case study, genetic expert service.**

A regional Victorian paediatrician contacted the genetic expert service to understand the process for directly requesting funded exome tests, rather than referring patients to genetics. During a 17‐min call, the genetic counsellor guided the paediatrician through the online test request portal, fulfilling Medicare funding criteria. Following this interaction, the paediatrician successfully placed a request for a second patient.

From April 2024 to February 2025, the teaching clinic had 62 appointments with 39 patients. Thirteen paediatricians attended the clinic for experiential learning and nine set the following learning goals:

1. Observe a consent discussion, gain skills to consent families independently for both genomic and other forms of paediatrician ordered genetic testing (e.g., microarray) (*n* = 7).
2. Gain experience with result disclosure, particularly variants of unknown significance (*n* = 5).
3. Learn how to access genomic testing as a paediatrician (understand practicalities of test ordering) (*n* = 3).
4. Better understand eligibility criteria (*n* = 2).
5. Learn to read exome reports and interpret results (*n* = 2).
6. Skills in conducting a dysmorphology assessment (*n* = 1).
7. Learn how to take a targeted genetics family history (*n* = 1).
8. Gain specific language tools for explaining genomic testing and diagnoses to families (*n* = 1).
9. Understand how undergoing a genomic test may help patients and families (*n* = 1).
10. Gain more confidence to order exomes independently (*n* = 1).

Paediatricians highlighted the value of experiential learning in interviews:

It was good to be able to sit in the room with everybody and have a simulated consult with them supporting me so I could get that hands‐on experience. I feel a lot more confident, and I didn't think twice about doing it (requesting funded tests) yesterday. (Paediatrician 5)

Under the RE‐AIM framework, evaluating adoption includes describing settings and practices in which the intervention has been adopted. Using patient postcodes we observed changes in test request distributions over the intervention period. File S6 shows maps overlayed with tests per 10 000 children and locations of awareness raising activities.

### 3.6 Implementation

On average, website users accessed 2.51 pages per visit, demonstrating engagement in the pages. They also spent 3:24 min actively navigating around the pages, above the industry standard of 2–3 min. The many link clicks and file downloads show users found the content helpful. The top three resources accessed/downloaded from the website were: laboratory consent forms (*n* = 116); the link to the laboratory online ordering system (*n* = 108) and the laboratory genomic testing factsheet (*n* = 85).

It's (the website) really great… It was worth every minute I spent on it and at some stage I might go back and have a look at things again just to refresh myself. (Paediatrician 6)

Interviews with the genetic experts providing the consultation service described their support as being practical regarding eligibility and request processes rather than the anticipated support about talking with families and the expertise a genetic counsellor can provide.

I thought that there would be more paediatricians contacting the service… I thought that they'd be more interested in the genetic counselling side of things, like the consent… (Genetic Expert 1)

However, they also described some of the psychosocial issues that might arise in a genetics‐related conversation that may be unfamiliar to a paediatrician

…paediatrician called me up quite stressed because there was a family dynamic issue. Parents were divorced and they didn't want their information passed on to the other person… I think maybe that's where they would need [genetic counselling] help. (Genetic Expert 1)

In interviews, paediatricians talked about using the website and consultation service to discuss specific clinical questions and determine if testing is the right option for their patient.

The fact that we can actually speak with a genetic counsellor through the website, that makes it easier, and we don't feel like we're, overdoing or being silly over ordering things. (Paediatrician 3)

The teaching clinic was well received by attending paediatricians who used it to gain skills to apply knowledge.

They catered towards my learning goals on how to run the consultation for testing… Before the patients got there, we had a discussion about the various resources and the language to use. We had some pictures out to discuss outcomes of genetic testing. And I gave them an update about my patients … that was really useful for both of us. For both the patients I went through the consult and practiced how to talk about genes and also talk about what whole exome sequencing is and the possible outcomes of doing the test. It was really good to do that and to have practiced before. (Paediatrician 5)

In addition to learning skills, it was normalising to observe that even genetic experts find cases and consultations challenging at times.

That was really helpful for me to know that the geneticists and the counsellors are wrestling with the same issues that we're wrestling with … that was helpful for me to know, it's not just me. I did come away with some good ideas for me in my practice. (Paediatrician 9)

A further example of the experiential learning gained through the teaching clinic is provided in Box 2.

BOX 2 **Case study, teaching clinic.**

A paediatrician attended the teaching clinic with two patients, aiming to improve communication and consent skills for genomic testing. In a pre‐clinic meeting, they reviewed cases and resources, and role‐played conversations. During appointments, the paediatrician led consent discussions using newly learned techniques. Following this experience, the paediatrician reported ordering funded exomes for other patients in their practice.

### 3.7 Maintenance

Table 1 shows over 80% of surveyed paediatricians anticipate using Medicare‐funded genomic tests as a regular part of their practice. Case studies in Boxes 1 and 2 illustrate how initial support leads to subsequent test requests.

Test requests increased over the period of the study (relative percentage reported): in the first 11 months after the website launched, and while active awareness raising was underway, paediatrician requested tests increased by 227% compared with baseline (11 May 2023 to 10 April 2024). From 11 April 2024 to 3 March 2025, during the period where the active study activity was the teaching clinic, there was a further 29% increase in test requests, compared with the previous time period.

It is important to note, however, that test request numbers alone do not fully reflect the impact on paediatricians' confidence and skills, or their future intent to request tests as described below.

I haven't ordered yet, but I have thought about it and it's something that I, the next time I see (patient), we'll talk through it. (Paediatrician 8)

## 4 Discussion

This study describes the co‐design, implementation and evaluation of interventions to support paediatricians in requesting funded genomic tests for their patients. The interventions were designed for paediatricians, by paediatricians.

Our findings demonstrate that targeted support can enhance paediatricians' confidence and begin to shift their practice towards directly requesting funded genomic testing for eligible patients. We observed increased confidence across all process steps from baseline to 1 month after website access. Interestingly confidence in informational and knowledge areas remained elevated at 3 months. However, confidence in skill‐based processes tended to return to baseline levels.

This pattern suggests that while knowledge transfer through resources like the website is valuable, it needs to be complemented by skills‐based experiential learning for paediatricians to develop comprehensive confidence in their practice. This observation aligns with Bloom's taxonomy, which posits that education transferring knowledge (as provided by the website) represents the lowest order of complexity [8]. Moving to independent practice requires applying knowledge, which is best facilitated through experiential learning opportunities [9, 10]. Our recommendation would be for paediatricians to gain experience that could be supported as they build confidence by contacting a service such as the liaison service, or participating in a teaching clinic. In the United Kingdom, there is work in progress to also provide online resources, curated to be relevant to the national context, for clinicians, including paediatricians, to support practice. Examples include the GeNotes resource [11] and the Genomics Education Programme. While such resources, ours included, are a part of the solution, a recent survey of paediatricians in the United Kingdom indicated that to improve their confidence to practice, support from clinical genetics services, direct experience and support from an experienced colleague are needed [12].

Our evaluation revealed that passive awareness‐raising methods, such as newsletter announcements, had limited impact on intervention access, shown by email address provision trends in Figure 1. In contrast, active awareness‐raising through clinic and department meetings, with a paediatrician and genetic counsellor present to answer questions, proved more effective.

The genetic expert service intervention leveraged the existing clinical genetics laboratory liaison service, and the evaluation reported in this article reflects only data generated by paediatricians who visited the website or attended a study awareness‐raising event and were directed to the expert service: there were few accesses recorded. There is no formal evaluation of the liaison service available; however it is a busy service and it received additional staff resources for the duration of this study. Therefore, it is a limitation of our study that a full picture of the usage of such a service is not available to report; however, the qualitative evaluation has revealed the impact the service has on promoting confidence and skills for paediatricians. This component of the intervention was delivered in parallel with the website and the teaching clinic: 222 paediatricians accessed the website and 13 came to the teaching clinic—therefore it is possible that those other avenues were meeting the support needs and contacting the consultation service was not required. Paediatricians reported that the support received helped them overcome barriers and equipped them to make requests for other patients. This service remains available through the clinical genetics laboratory to support paediatricians in the future.

It is important to note that while changes in test request volumes might seem an obvious measure of success, we considered this a secondary outcome due to various factors influencing testing decisions. For example, during the period we were evaluating, paediatricians may have become more confident and willing to order funded testing; however they may not have had an appointment with an eligible patient. Longer‐term evaluation would further assess the impact of these interventions on paediatrician practice; it is a limitation of our study that test audit data were only available up to March 2025 to use for the evaluation of the impact of the intervention; therefore the impact we report may be an underestimate.

The interventions were implemented in a real‐world context where requests for funded genomic tests gradually increased from a low baseline [4]. Therefore, we claim a contributory role in the increase in test volumes rather than attributing changes solely to the interventions. Nevertheless, our mixed‐methods evaluation provides valuable insights into the success of the interventions particularly on confidence and skill development through experiential learning, explanations for usage patterns, and anticipated future use, offering a foundation for ongoing efforts to support paediatricians in genomic testing practices. Response rates for the evaluation surveys were low, which is expected in survey studies with medical health professionals; a previous national survey of genetics practice we conducted elicited responses from approximately 1.2% of medical specialists registered with the Medical Board of Australia [13]. Knowing that response rates to surveys would be low, our mixed‐methods approach including interviews and website use metrics facilitated an informative evaluation.

Understanding the impact of the interventions on the profession of paediatricians in Australia is challenging in that there is no register that captures information regarding the specifics of practice of paediatricians; not all paediatricians see children who would be eligible for funded exome tests (or only have a few on their list). We know that relevance to practice is a driver of interest and engagement in upskilling activities [14] and therefore anticipate that our denominator for the reach of impact of these interventions is not the total number of paediatricians in Victoria, but is the number of paediatricians for whom exome testing is or will be in the near future, a part of their practice. This number is unknown.

In conclusion, this study has shown that practice change can be supported with interventions designed by clinicians, and has wide reach when awareness is raised through diverse channels. The co‐design of interventions meant that paediatricians had access to the mode of support they desired, in a way that was accessible to them. Even when an intervention is co‐designed, such as the expert consultation service, a pilot should be undertaken to inform how success will be measured and consider quality of impact as well as usage. Paediatricians from diverse clinical and geographical contexts engaged with the interventions in this study to support their practice which resulted in the wider (geographical spread) use of genomic tests for children with developmental delay and unique body features.

## Ethics Statement

This study has approval from the University of Melbourne, HREC ID 25209 (co‐design workshops); the Royal Children's Hospital Melbourne, HREC 94607 and Monash Health, Melbourne HREC SSA/94607/MonH‐2024‐437 148(v2).

## Conflicts of Interest

The authors declare no conflicts of interest.

## Supporting information


**File S1:** jpc70237‐sup‐0001‐FileS1.docx.

**File S2:** jpc70237‐sup‐0002‐FileS2.docx.

**File S3:** jpc70237‐sup‐0003‐FileS3.docx.

**File S4:** jpc70237‐sup‐0004‐FileS4.docx.

**File S5:** jpc70237‐sup‐0005‐FileS5.docx.

**File S6:** jpc70237‐sup‐0006‐FileS6.docx.

## Data Availability

The data that support the findings of this study are available from the corresponding author upon reasonable request.
