# Supplementary material for: Exomes in Paediatrics: Co‐Design and Implementation of Interventions to Support Paediatricians to Provide Genomic Care
Source: J Paediatr Child Health. 2025 Nov 20;62(1):97–105. doi: 10.1111/jpc.70237 (PMC12800879; doi:10.1111/jpc.70237)
Supplement: Supplementary file 1 — File S1: jpc70237‐sup‐0001‐FileS1.docx. [file JPC-62-97-s001.docx]

**Supporting Information File 1: Co-design process and outputs**

B. Dawson‐McClaren, M. Martyn, E. Weisz, et al., “Exomes in Paediatrics: Co‐Design and Implementation of Interventions to Support Paediatricians to Provide Genomic Care,” *Journal of Paediatrics and Child Health* (2025): 1–9, https://doi.org/10.1111/jpc.70237

The co-design approach taken

Experience-based co-design was used to develop intervention strategies aimed at addressing barriers for paediatricians to order funded exome for eligible patients (Bate P and Robert G, 2006). In this approach, co-designers are engaged in various participatory activities including journey mapping, storytelling, identifying touch points, prototyping interventions, and identifying barriers and enablers.

The three stakeholder groups - paediatricians, parents and genetic health professionals – engaged in the co-design approach represent key users of any service or system in which eligible children can access the relevant funded genomic tests; in this project the context is Medicare funded exome tests for children with childhood syndromes. With our focus on intervening to improve access we centred the paediatrician stakeholder group. That is, we sought to develop and implement strategies to support paediatricians to identify eligible patients, develop skills and confidence to conduct consultations, and have practical supports to complete test request steps (Dawson-McClaren B., et.al, 2025).

Parents provide important perspectives even when paediatricians are the focus of interventions because their experiences of the ways of practicing that paediatricians will develop must be anticipated to ensure that there is a fit with their expectations and preferences. Similarly, genetic health professionals will interact with paediatricians’ practice of ordering funded genomic tests through each of the steps identified in our previous work (Dawson-McClaren B., et.al, 2025) namely: co-consultations, providing advice regarding test suitability, offering practical support, and likely seeing families in future appointments if subsequent investigations or discussion beyond the scope of the paediatrician’s practice are warranted.

In summary, we used a series of online workshops with the stakeholder groups, separately, to gather experience-based information, and design principles for potential interventions, that, analysed alongside our previous dataset of interview with 26 individual paediatricians, guided our informed development of interventions to trial in a real-world setting. The evidence-based co-design process makes the user experience accessible to those charged with designing, in our case, the interventions for paediatricians.

The primary input for co-design workshops was the findings from our previous interview study (Dawson-McClaren B., et.al, 2025) with 26 paediatricians which explored in depth their experiences of genomic investigations for children in their care, barriers encountered or anticipated when ordering funded tests, and suggestions for interventions that may address the barriers. We also interviewed 5 parents, unpublished data, to gather initial information on relevant touchpoints that would serve as input for co-design workshops.

The co-design workshops were facilitated by the co-design and implementation effectiveness team within the Methods and Implementation Support for Clinical and Health Research Hub (MISCH-IEF). This team operate a co-design living labs program that has established processes and methods for experience based co-design.

Co-design cycle 1: The aim of these workshops was information gathering around needs and potential interventions. “Touchpoints”, the key moments in a journey/process or the places people come interact with the topic, were used to guide the discussion.

Co-design cycle 2: These workshops and meetings aimed to 1) develop design principles for interventions proposed in cycle 1, 2) commence direct development such as reviewing potential existing resources that could be on a website and practical considerations to implement a consultation service.

Participant eligibility and recruitment was as follows:

- Parents. Invited to respond to an expression of interest advertising the study through relevant support groups. Parents were eligible if their child had previously had genetic testing before the age of 10, with a presentation of developmental delay; and their child has a paediatrician involved in their care.
- Paediatricians. Invited because they had previously participated in a research interview and gave permission to be contacted for further activities. In the previous interview study, there were 26 paediatricians with the following characteristics: 16 practiced in metropolitan Melbourne, 10 in regional sites across Victoria. Nine were in private practice only, the rest practiced in public hospitals of which 8 were at a site with an in-house clinical genetics service and 9 at a site with a visiting clinical genetic service.
- Genetic health professionals. Invited because they were known by the research team to work in the area of exome testing for childhood syndromes. These included clinical geneticists, genetic counsellors and laboratory staff.

Due to the likely challenges of scheduling group events, participants were provided with the dates of all the activities for their stakeholder group and invited to join any they were available for.

Details of co-design cycles and participants

#### Cycle 1: October-November 2022

Three workshops were held over three weeks for either parents (n=6); paediatricians (n=3, two in private practice and one working at a tertiary public hospital) or genetic health professionals (n=4, representatives from two genetic services in Victoria). Each workshop was held online, for two hours in duration. All participants were invited to take part through an emailed invitation and provided with a participant information statement about the study.

Cycle 1 workshops elicited experience-based discussion to identify and explore touchpoints in which these users have or anticipate interacting with a step in the process for a child to access funded exome testing. Using participatory approaches, participants were encouraged to use storytelling and engage with interactive tools.

Touchpoints: The first activity of each cycle 1 workshop presented participants with proposed touchpoints relevant to their stakeholder group. These were developed by the research team, informed by the prior interview study, and presented on a journey board representing a child/parent’s journey through genomic testing. Participants were asked to add anything that was missing, from their experience of being a parent or supporting parents through genomic testing. Thinking about positive and negative aspects was encouraged.

Storyboard: The second activity asked participants to ‘complete the story’ of a fictitious family seeking genomic testing for their child. The discussion was about ideas on what supports might help the family and health professionals involved in the journey, and to think about how other stakeholder groups might perceive those support ideas. This activity in particular began to generate key principles for the co-designed interventions and unpacked the priorities the groups had, revealing similarities and differences.

In addition, participants were invited to imagine a co-designed resource existed for paediatricians and to discuss what it looks like, who was involved in making it and what happens next with it. This activity further generated ideas around what the co-designed outputs would be and extended the discussion to consider what they would look like, feel like and function like. Once again participants were invited to draw on their experience and also consider how their contributions might be experienced by other stakeholder groups.

#### Cycle 2: February 2023

In order to develop the ideas for resources further, two workshops were held on sequential days in February 2023, one with paediatricians (N=3, one from cycle 1) and one with parents (N=4, two from cycle 1).

Genetic health professionals participated in in-person 2 hour meetings over two weeks focussed on the intervention dependent on their ongoing participation: development of a phone line staffed by genetic experts. The first meeting was held twice to optimise participation from genetic health professionals from the two major genetic services in Victoria while all (N=6) were able to attend the second meeting.

Outputs of co-design workshops

We present firstly key findings relating to the touchpoints and storyboards from each of the workshops – effectively the needs, challenges and opportunities - and then describe findings relating directly to potential interventions.

##### Touchpoints and storyboards

***Paediatricians’ touchpoints*** (Figure 1) related to a sense of their limitations in knowledge and understanding of process steps, including consent discussions, completing ordering tasks, sample collection and relaying results. The touchpoints introduced from the prior interview study (shown in blue) were endorsed in this workshop.

The funded test can be ordered by a paediatrician, however for the test to be reimbursed they must consult with a clinical geneticist. In this workshop, paediatricians discussed challenges with timely access to a geneticist, resulting in decisions to instead refer patients to clinical genetics service for testing to be arranged. They preferred real time access to and support from clinical geneticists and genetic counsellors. Paediatricians were also concerned about the wait time their patients might experience after a result, if they were to seek genetic counselling follow up.


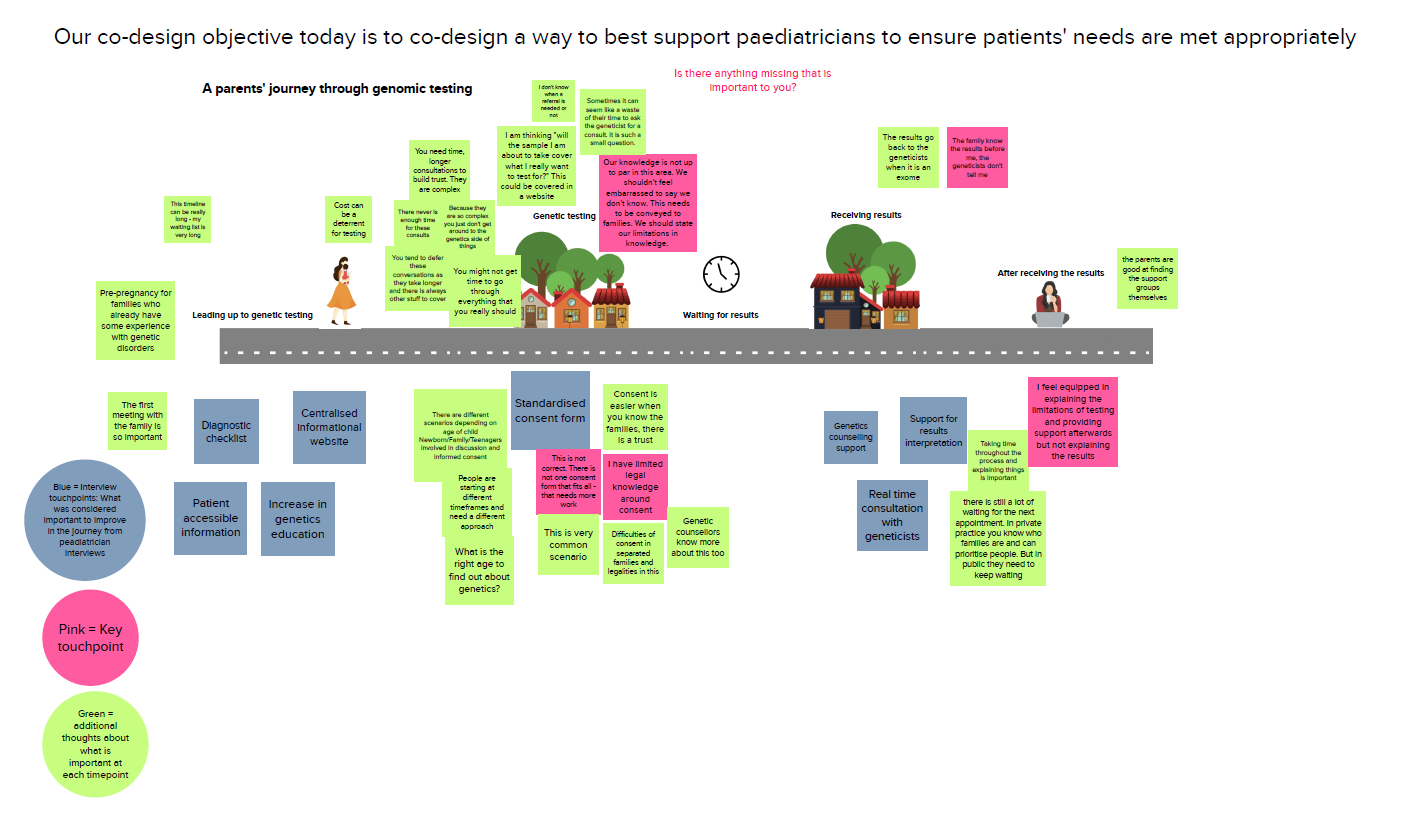


**Figure 1:** Activity 1 Paediatrician co-design workshop, discussion of touchpoints. Blue represents touchpoints identified in the previous interview study and were presented to paediatricians to initiate discussion. Pink items were additional key touchpoints added during the workshop. Green items represent additional thoughts of the workshop attendees as they discussed touchpoints.

The ‘story board’ – where paediatricians completing the story of a fictitious family –is shown in Figure 2. The pink notes were added by paediatricians to describe the support, information or clarification they would like and interventions they propose to address related to the various touchpoints.


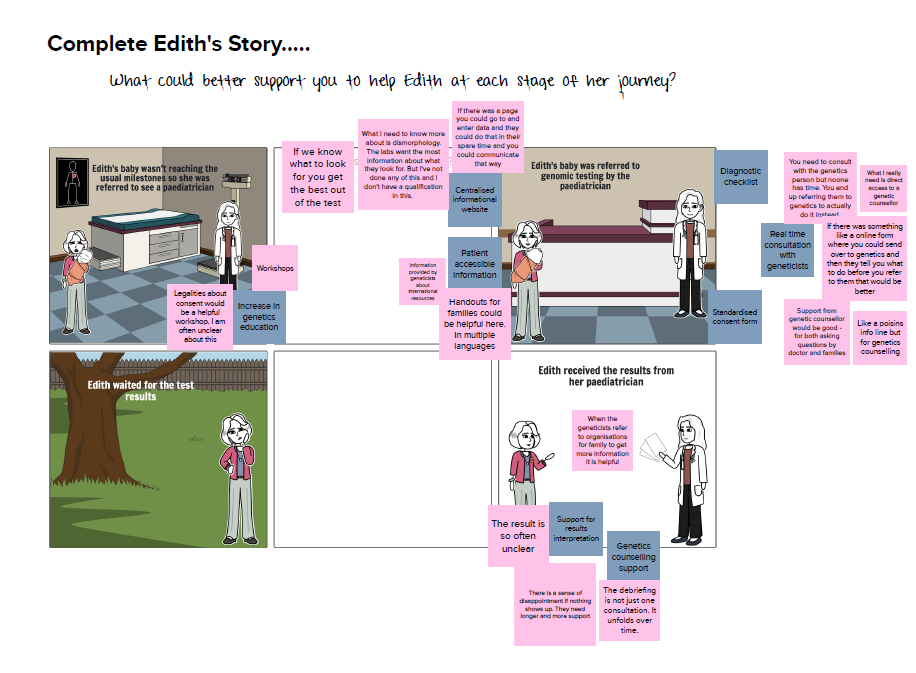


**Figure 2:** Activity 2 Paediatrician co-design workshop. Completion of a story board of a future state of how to best support families when providing care. Blue items represent touchpoints paediatricians identified, pink items are notes paediatricians added to those touchpoints to clarify their support and information needs.

**Parents** added touchpoints primarily at the later stages of testing and what happens after a result (Figure 3). They were seeking appropriate and deliberate referrals in the form of a ‘follow up plan’ that involves counselling, support organisations, genetic counselling and genetic services. Parents did not expect that a paediatrician would be expert in genetic conditions and their implications but did expect that they would ensure family and patient needs were met by being able to offer support and refer appropriately.


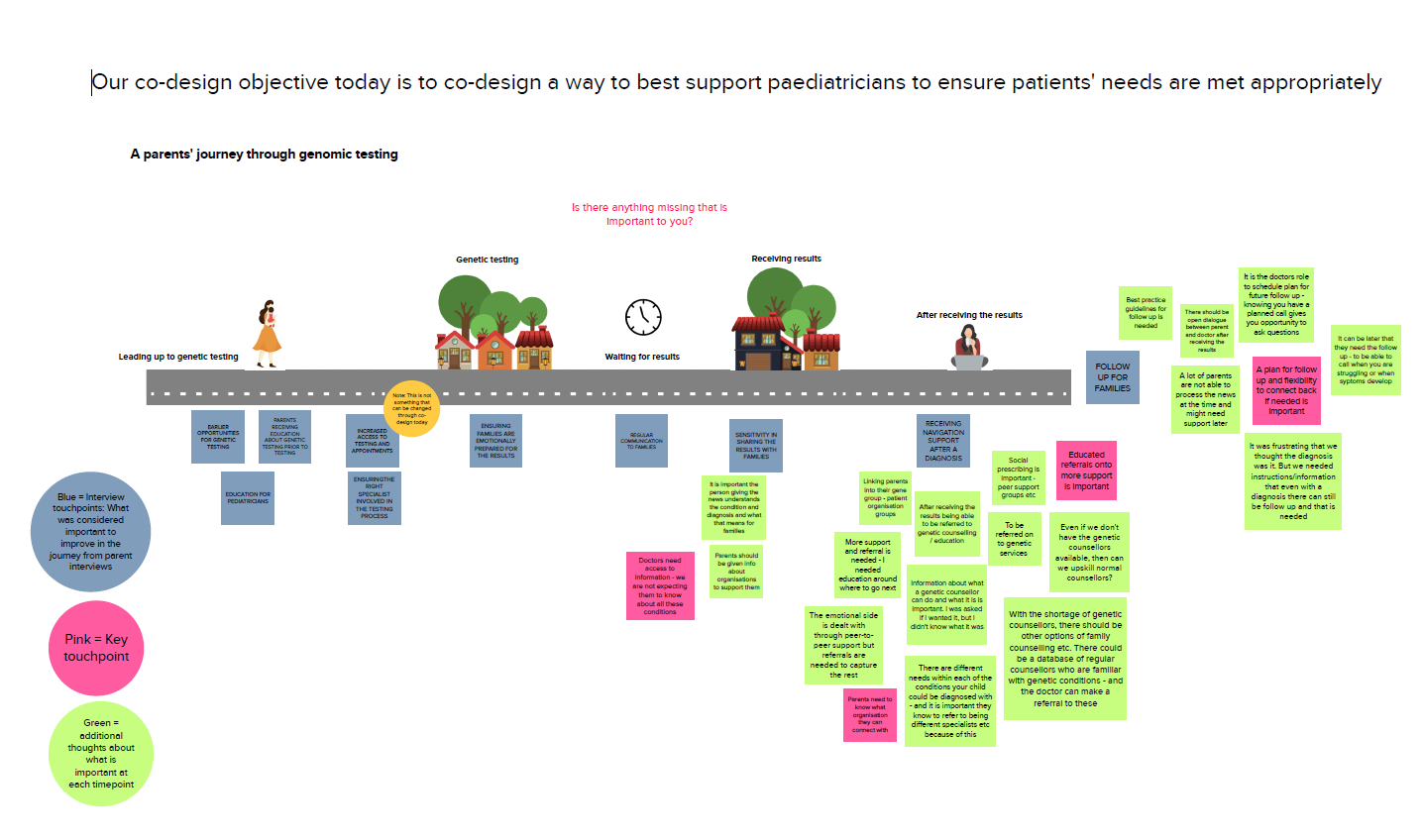


**Figure 3:** Activity 1 Parent co-design workshop. Blue items represent touchpoints raised in interviews, unpublished, prior to the co-design workshop and served as input for discussion in the co-design workshop. Pink items were additional key touchpoints proposed in the workshop with green items providing additional thoughts and considerations related to touchpoints.

The storyboard completed by parents (Figure 4) added comments regarding the information, supports and expectations at each stage.


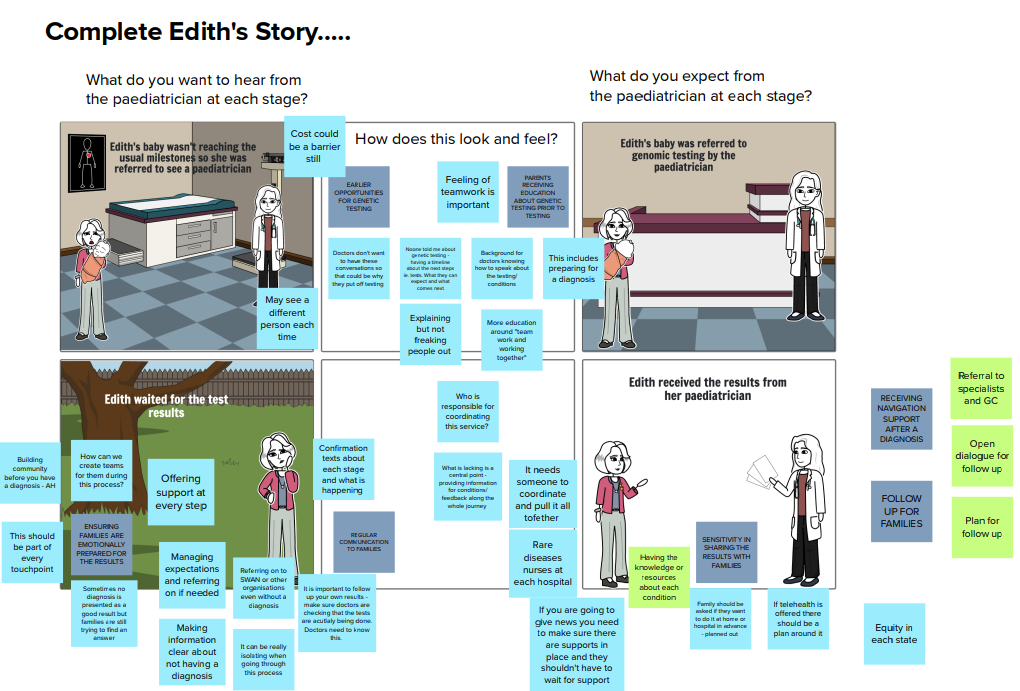


**Figure 4:** Activity 2 Parent co-design workshop. The completed story board shows touchpoints in dark blue with light blue items providing additional clarification of supports and information needs relevant to stages in the journey. Green items were important next steps in the journey, specifically identifying follow up needs and plans.

The **genetic health professionals** identified touchpoints (Figure 5) relating to process steps, with a focus on how they may support and upskill paediatricians along the journey. They discussed the scope of roles and expectations of who does what. Patient consent was used as an example where genetic health professionals had specialized skills, knowledge and training that paediatricians may not. They recognised that paediatricians have irregular opportunities to apply knowledge and skills into action to develop confidence to practice and indicated a desire to support them.


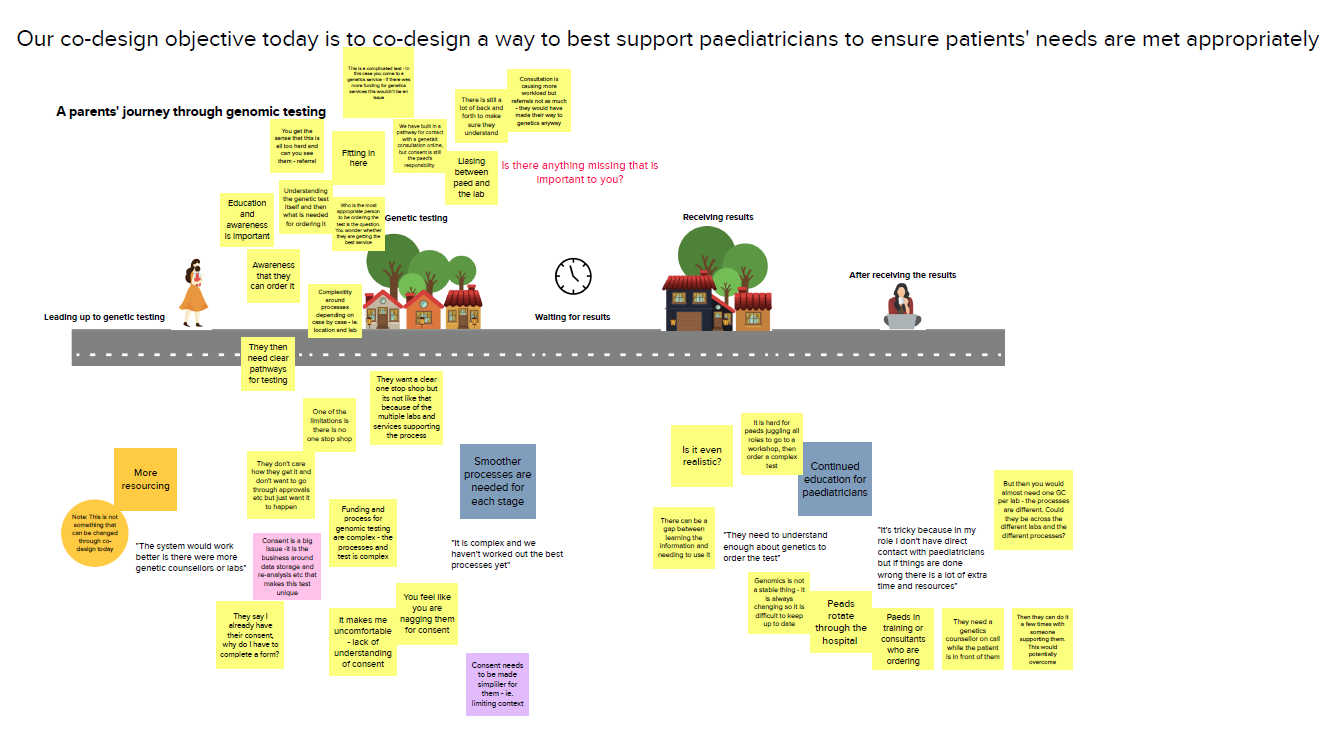


**Figure 5:** Activity 1 Genetic health professionals co-design workshop. The blue items were the two touchpoints participants identified as most relevant, with yellow items representing the discussion points raised around these touchpoints.

The story board completed by genetic health professionals resulted in an ideal practice comment box for each stage of the journey (Figure 6).


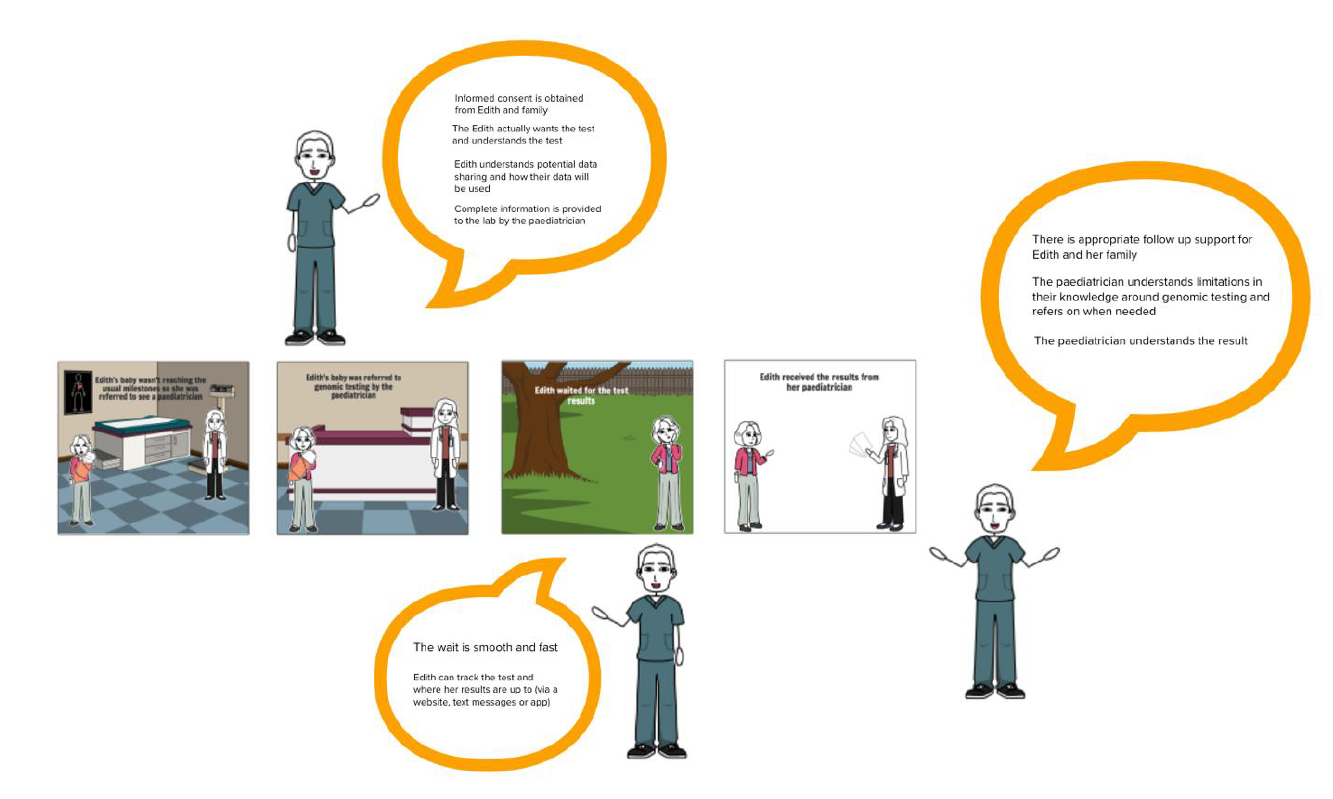


**Figure 6**: Activity 2 story board genetic health professionals. Ideal practice at the journey stages was summarised in comment bubbles.

##### Priorities to support paediatrician practice

A centralised website

Both paediatrician and genetic health professional groups suggested information provision^[[1]](#footnote-2)^ through a centralised website strategy that is a website that services as a ‘landing site’ that is easy for paediatricians to remember and access. Different modes of information were proposed and key requirements identified including:

- Learning resources and options for understanding genomic testing and conditions relevant to the paediatric population
- The details of a phone line for genetic staff support, or a way to ask questions (this was further discussed, with detail described below)
- Information about genetic services and options for patient referrals
- A step-by-step guide about testing for paediatricians and patients
- Information and links on how to get support
- Frequently Asked Questions

A list of local, national and international sites and resources was compiled (not shown) and affirmed in the cycle two workshops as appropriate sources with a preference for local or Australian-based information.

Parents requested that paediatricians be provided with a database of support organisations and information about genetic conditions. They also sought resources that provided information when a donor had been used for conception, navigating complex family scenarios such as when a member does not want to be part of the testing process, and National Disability Insurance Scheme resources for families with genetic conditions.

Table 1 provides a comparison of the essential features of the website that were proposed during co-design and what was included in the resultant intervention.

A consultation phone line

Both health professional groups suggested a phoneline^[[2]](#footnote-3)^ for paediatricians to access real time consultations with clinical geneticists during the testing process. Similar services were given as examples supporting other areas of practice. Further discussion of this concept revealed that a genetic counsellor responding to the phone line may be more practical to implement and could provide coaching and support as paediatricians navigate their initial experiences with ordering funded tests.

Experiential learning

The previous interview study raised the possibility of co-consultations^[[3]](#footnote-4)^ as an experiential learning opportunity with a genetic health professional to observe and learn from them how they conduct an appointment (Dawson-McClaren BJ., et. al, 2025). The feasibility of this form of intervention was not possible at the time of the co-design activities so was not discussed. However, after the study paediatrician EW was employed in March 2023 this proposal was further developed resulting in the commencement in April 2024 of the paediatric exome teaching clinic providing experiential learning for paediatricians.

**Table 1:** Comparision of implemented interventions to essential website features requested in co-design process

| **Essential website features** | **Met or not by implemented intervention(s)** | **Description** |
| --- | --- | --- |
| Meet needs of families as well as health professionals | Yes | Content written by a paediatrician and a communications specialist from Melbourne Genomics Health Alliance and pitched at both these audiences where practical. Embedded through the website are resources for paediatricians and for families.  The landing page also has a direct link to a sub-page providing patient and community specific resources including printable materials. |
| Search feature | Partially | The parent website for Melbourne Genomics has a search function that returns results from this paediatrician resource as well as other linked projects.  The use of a navigation pane and headings was intended to reduce the need for a more sophisticated search feature |
| FAQ | Partially | There is no dedicated FAQ section however the headings of the content in the step-by-step guide are phrased as questions in many instances. |
| Step by step guide | Met | The landing page provides three options, one of which is a step-by-step guide. The guide has a navigation pane with the steps set out chronologically. |
| Request and consent forms available | Met | In addition to specific laboratory consent forms, order guides and order systems, there are examples provided of generic consent forms, key points to cover in a consent conversation and resources that can be used during the consultation including Plain English and Easy English explainers |
| Consent and request forms auto populated | Not met | No single electronic patient management system exists and integration of systems beyond scope of this project (feasibility, time, budget constraints). |
| Family pedigree drawing tool | Partially | Licensing and integration requirements to develop or adopt an existing tool was beyond scope (time and budget constraints).  Under the heading of completing the test request form, there is a section regarding preparing what you need. A link is provided to a website giving instructions on drawing pedigrees, and an article regarding writing inclusive and affirming pedigrees. |

**Acknowledgements**

All authors would like to acknowledge MISCH (Methods and Implementation Support for Clinical Health research platform), Faculty of Medicine, Dentistry and Health Sciences at University of Melbourne for its technical support to conduct the co-design activities described in this document, that is a supplementary file to “CITATION OF ARTICLE WHEN AVAILABLE”

**References**

Bate P, Robert G. Experience-based design: from redesigning the system around the patient to co-designing services with the patient. Qual Saf Health Care. 2006 Oct;15(5):307-10. doi: 10.1136/qshc.2005.016527. PMID: 17074863; PMCID: PMC2565809

Dawson-McClaren B, Martyn M, Ince J, Jan A, Brown NJ, Fahey MC, Crellin E, Gaff C. Opportunities and challenges for paediatricians requesting funded genomic tests for children. Eur J Hum Genet. 2025 Sep;33(9):1173-1179. doi: 10.1038/s41431-025-01864-3. Epub 2025 May 7. PMID: 40335663; PMCID: PMC12402192.

Powell, B.J., Waltz, T.J., Chinman, M.J. et al. A refined compilation of implementation strategies: results from the Expert Recommendations for Implementing Change (ERIC) project. Implementation Sci 10, 21 (2015). https://doi.org/10.1186/s13012-015-0209-1

1. This suggestion aligns with established implementation strategies from the compilation generated by the Expert Recommendations for Implementing Change project (Powell BJ., 2015). Specifically: “Distribute educational materials: Distribute educational materials (including guidelines, manuals, and toolkits) in person, by mail, and/or electronically”. [↑](#footnote-ref-2)
2. From Powell BJ., 2015, this suggestion aligns with: “Centralize technical assistance: Develop and use a centralized system to deliver technical assistance focused on implementation issues”; “Provide ongoing consultation: Provide ongoing consultation with one or more experts in the strategies used to support implementing the innovation” [↑](#footnote-ref-3)
3. This suggestion aligns with “Shadow other experts: Provide ways for key individuals to directly observe experienced people engage with or use the targeted practice change/innovation”, (Powell BJ., 2015) [↑](#footnote-ref-4)
