# Supplementary material for: Exomes in Paediatrics: Co‐Design and Implementation of Interventions to Support Paediatricians to Provide Genomic Care
Source: J Paediatr Child Health. 2025 Nov 20;62(1):97–105. doi: 10.1111/jpc.70237 (PMC12800879; doi:10.1111/jpc.70237)

**File S2: Details of the website intervention content and structure**

B. Dawson‐McClaren, M. Martyn, E. Weisz, et al., “Exomes in Paediatrics: Co‐Design and Implementation of Interventions to Support Paediatricians to Provide Genomic Care,” *Journal of Paediatrics and Child Health* (2025): 1–9, https://doi.org/10.1111/jpc.70237

[www.paediatricgenomics.org.au](http://www.paediatricgenomics.org.au)

This website, co-designed with paediatricians, parents and genetic health professionals, sought to provide one place to go for paediatricians to access information and resources to support their practice. Content was derived from existing sources where available, with summary information written by the study paediatrician (EW).

Landing page


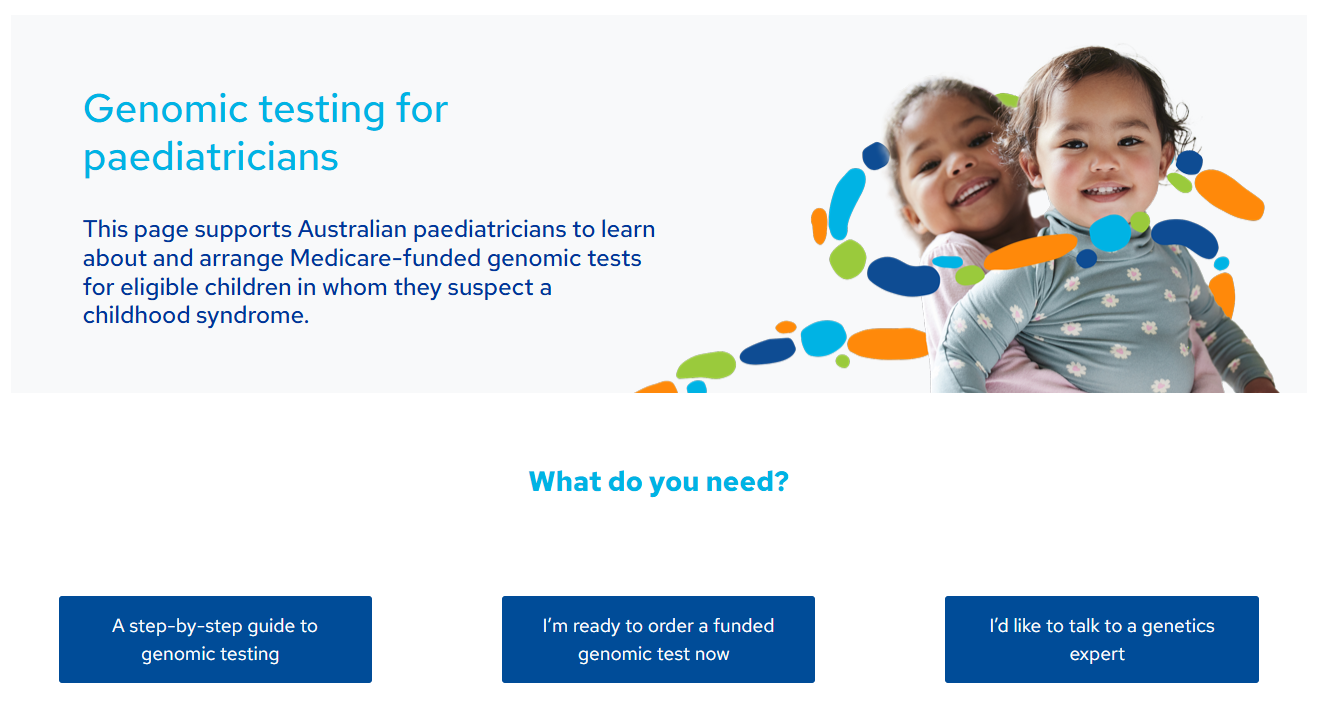


Step-by-step guide

During the study period, to evaluate the use of the intervention, instant access to this study page was granted after provision of name, email address and postcode of practice. This was to establish that users of the page were paediatricians, and to send evaluation surveys. This feature has now been removed.


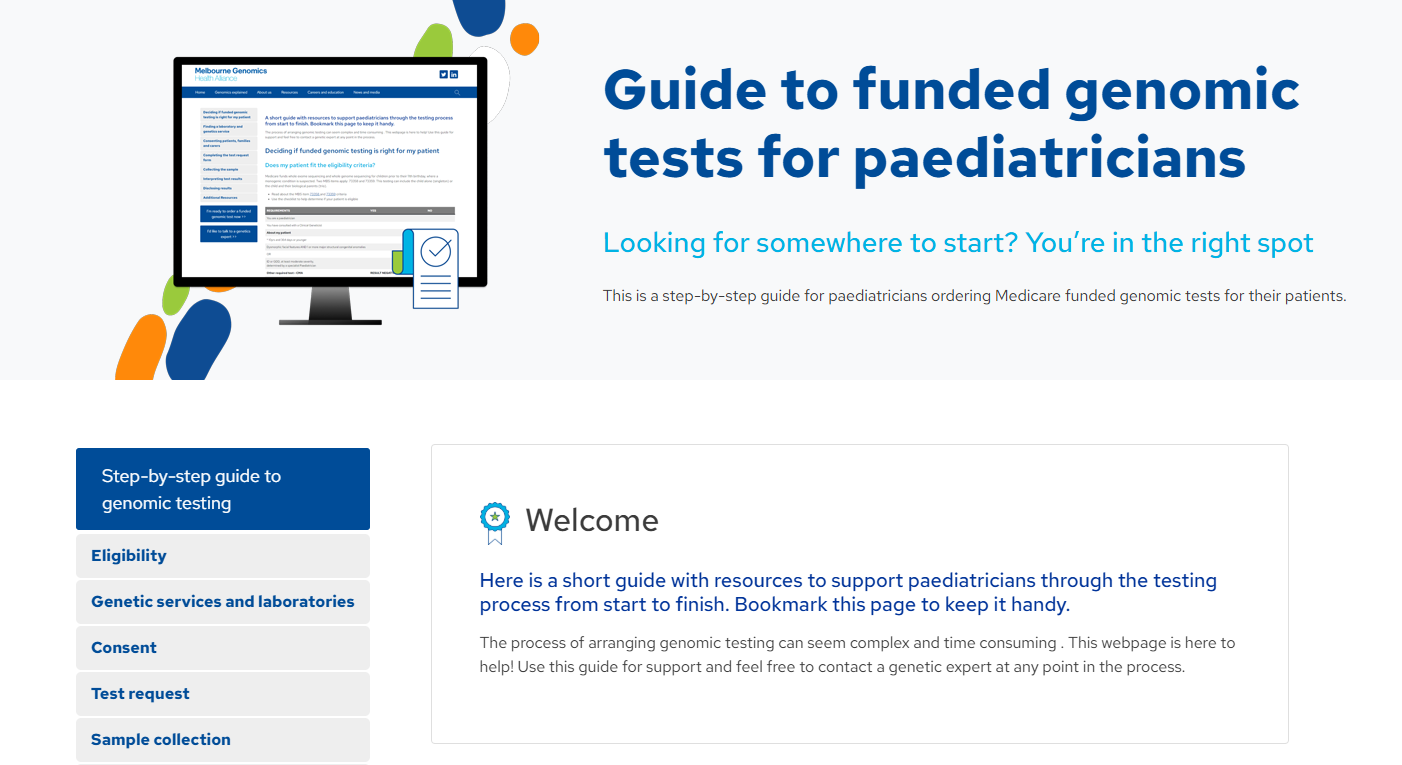


Videos of the study paediatrician


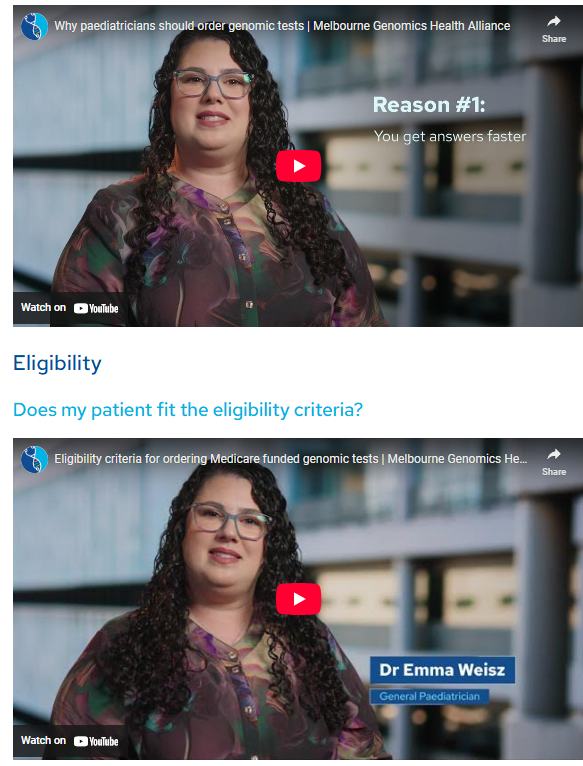


A checklist for patient eligibility


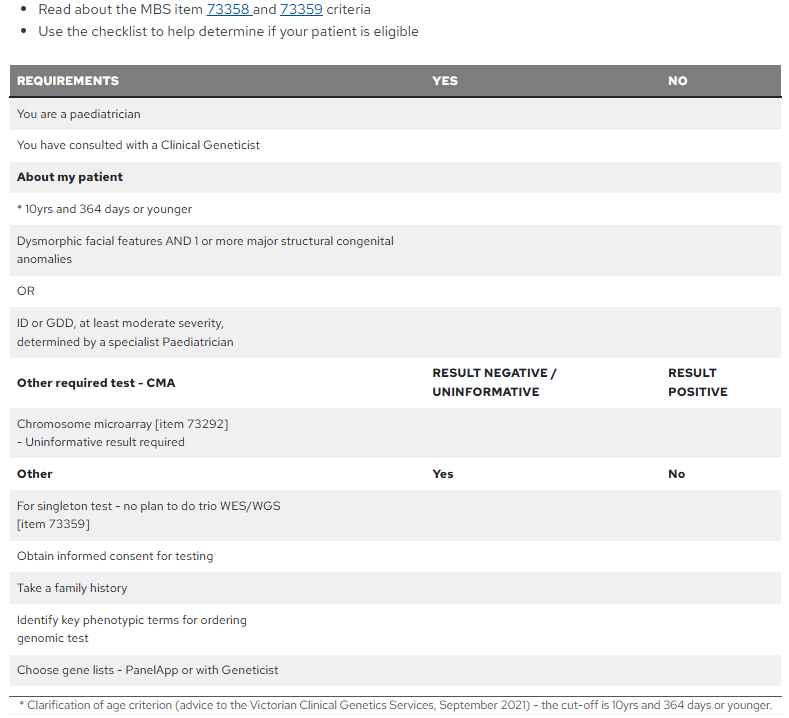


Navigating the step-by-step guide: each section provides an explanation and resource links


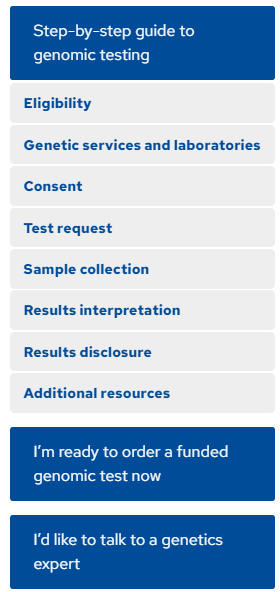


Genetic expert consultation service


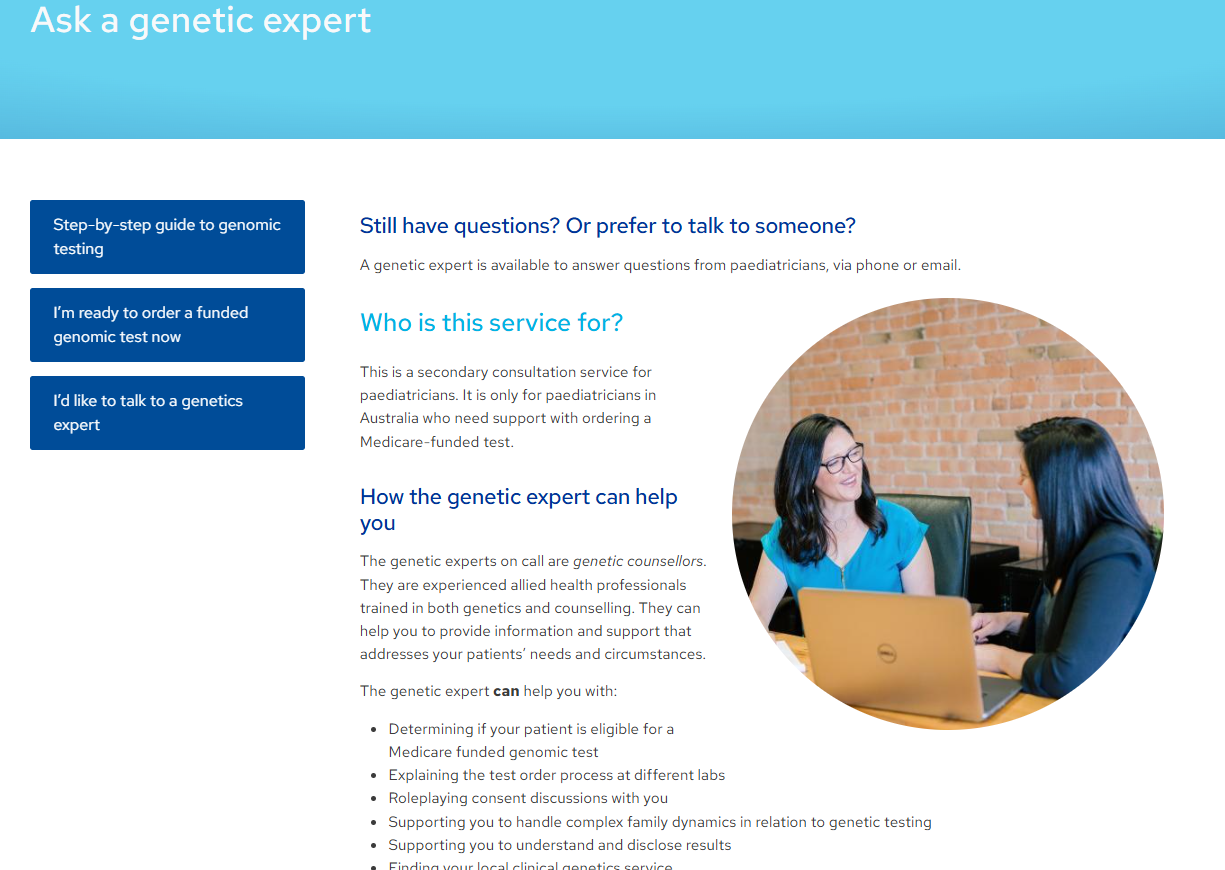


Direct link to genetic services and laboratories


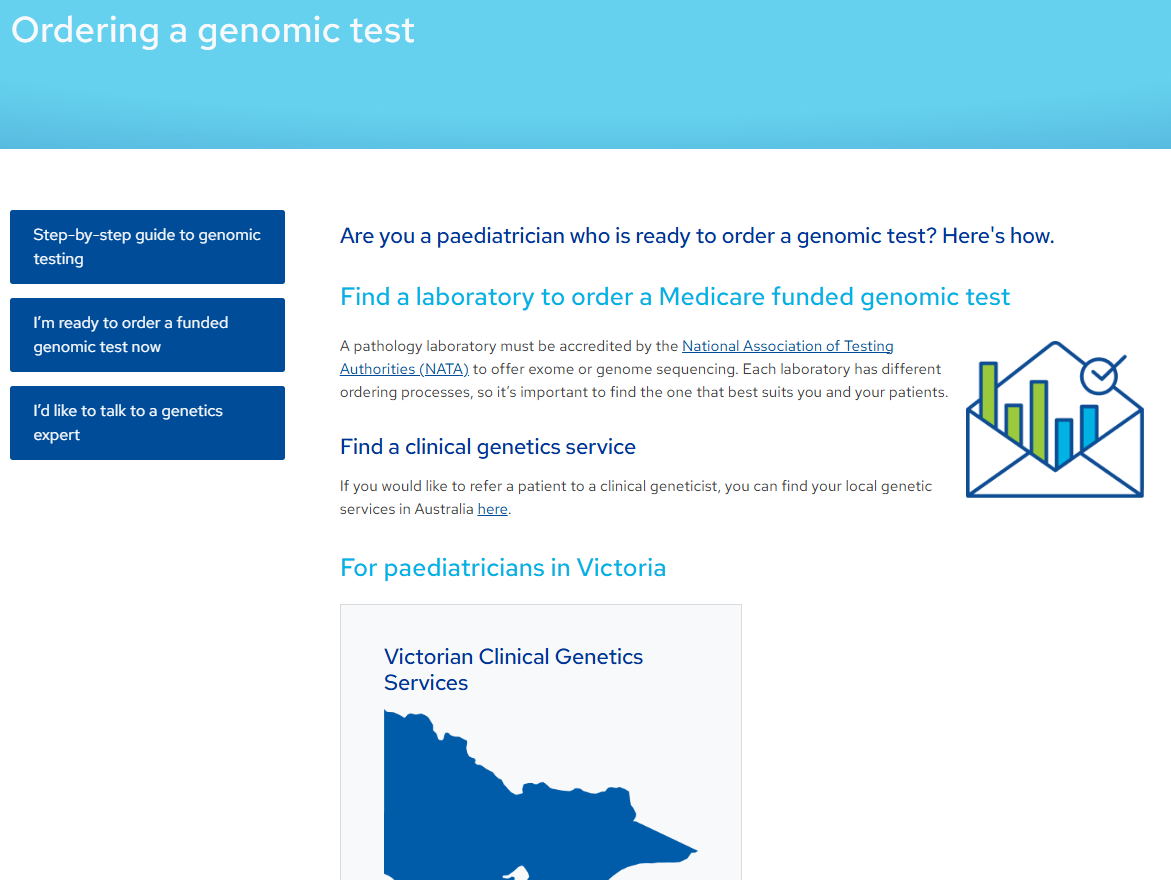

Supplement: Supplementary file 2 — File S2: jpc70237‐sup‐0002‐FileS2.docx. [file JPC-62-97-s006.docx]
