# Supplementary material for: Exomes in Paediatrics: Co‐Design and Implementation of Interventions to Support Paediatricians to Provide Genomic Care
Source: J Paediatr Child Health. 2025 Nov 20;62(1):97–105. doi: 10.1111/jpc.70237 (PMC12800879; doi:10.1111/jpc.70237)
Supplement: Supplementary file 3 — File S3: jpc70237‐sup‐0003‐FileS3.docx. [file JPC-62-97-s002.docx]

**File S3: The RE-AIM framework outcomes and the definitions used in this study.**

B. Dawson‐McClaren, M. Martyn, E. Weisz, et al., “Exomes in Paediatrics: Co‐Design and Implementation of Interventions to Support Paediatricians to Provide Genomic Care,” *Journal of Paediatrics and Child Health* (2025): 1–9, https://doi.org/10.1111/jpc.70237

In this study, we drew on the RE-AIM framework to guide the initial mixed-methods evaluation of the implementation of our multi-modal intervention.

The RE-AIM framework can be found here: [www.re-aim.org](http://www.re-aim.org)

It consists of 5 key outcomes: Reach, Effectiveness, Adoption, Implementation, and Maintenance; and is most commonly used as an evaluative framework for implementation of evidence-based interventions.

The authors of the framework recognise that not all outcomes will be measured, or fully measured in every project and support the use of mixed methods (e.g., surveys and interviews) to investigate these outcomes where possible, and/or expectations and perceptions of the outcomes. These initial assessments can inform future projects. Our study has used the RE-AIM framework in this way to evaluate the implementation, with the available data sources over the available study period.

In our evaluation, we refined the framework outcome definitions as specific research questions to be fit for purpose for our use in evaluating the implementation of our multi-modal intervention. Table Z1 shows the original framework definitions and our refined definitions.

**Table Z1**: RE-AIM framework definitions and refined definitions used in this study

| **RE-AIM outcome definition: www.re-aim.org** | **Refined definition, phrased as a research question** |
| --- | --- |
| REACH: The absolute number, proportion, and representativeness of individuals who are willing to participate in a given initiative, intervention, or program, and reasons why or why not. | REACH: at the individual level, did the interventions reach our target population of general paediatricians?  Our study could only report absolute number as well as reasons why or why not.  Data sources:   - Total number of paediatricians who were directly informed about the intervention - Total number of paediatricians who accessed the website |
| EFFECTIVENESS/EFFICACY: The impact of an intervention on important individual outcomes, including potential negative effects, and broader impact including quality of life and economic outcomes; and variability across subgroups( generalizability or heterogeneity of effects).  Further advice indicates that this outcome should measure a change in behaviour as a result of the intervention | EFFICACY/EFFECTIVENESS: What was the change (positive, negative, neutral) in paediatrician confidence and familiarity following intervention access?  Data sources:   - Surveys measuring confidence change over time - Interviews exploring how paediatricians confidence is changing |
| ADOPTION: (Setting levels) The absolute number, proportion, and representativeness of settings and intervention agents (people who deliver the program) who are willing to initiate a program, and why. Note that adoption can have many (nested) levels- for example, staff under a supervisor under a clinic or school, under a system, within a community. | ADOPTION: Did paediatricians, and across what settings, use the intervention(s)?  Our interventions did not require initiation at different sites however we could report on the use of the three interventions at different settings, and comment on the settings from which paediatricians practice by reporting postcode data of test requests and awareness raising activities.  Data sources:   - Website use metrics - Contact with the consultation service - Participation in the teaching clinic - Postcode analysis of test request audit data and of awareness raising activities |
| IMPLEMENTATION: At the setting level, implementation refers to the intervention agents’ fidelity to the various elements of an intervention’s key functions or components, including consistency of delivery as intended and the time and cost of the intervention. Importantly, it also includes adaptations made to interventions and implementation strategies. | IMPLEMENTATION: Were the interventions used as intended? E.g. what was used, how was it used and what wasn’t used?  The intervention was not delivered as a program therefore implementation evaluation is not directly measured but can be assessed by understanding how the interventions are used, to inform future projects.  Data sources:   - Website engagement metrics - Interviews regarding anticipated and actual use of the consultation service - Interviews regarding use of the teaching clinic to build practical skills |
| MAINTENANCE: At the setting level, the extent to which a program or policy becomes institutionalized or part of the routine organizational practices and policies. Within the RE-AIM framework, maintenance also applies at the individual level. At the individual level, maintenance has been defined as the long-term effects of a program on outcomes after a program is completed.  The specific time frame for assessment of maintenance or sustainment varies across projects. | MAINTENANCE: Has there been a change in confidence and practice (test requests) over time?  This study sought to measure individual level maintenance; individual change regarding confidence and practice.  Data sources:   - Application of knowledge and confidence as demonstrated by test request behaviour measured in case studies from contact with consultation service, and audit of test requests over time - Interviews with paediatricians |
