# Supplementary material for: Exomes in Paediatrics: Co‐Design and Implementation of Interventions to Support Paediatricians to Provide Genomic Care
Source: J Paediatr Child Health. 2025 Nov 20;62(1):97–105. doi: 10.1111/jpc.70237 (PMC12800879; doi:10.1111/jpc.70237)
Supplement: Supplementary file 4 — File S4: jpc70237‐sup‐0004‐FileS4.docx. [file JPC-62-97-s003.docx]

**File S4: Interview guides for evaluation interviews conducted with paediatricians and genetic experts**

B. Dawson‐McClaren, M. Martyn, E. Weisz, et al., “Exomes in Paediatrics: Co‐Design and Implementation of Interventions to Support Paediatricians to Provide Genomic Care,” *Journal of Paediatrics and Child Health* (2025): 1–9, https://doi.org/10.1111/jpc.70237

Paediatrician Interview guide

Thank you for making the time for this research interview. We will be talking about your experiences with genomic testing for your patients and specifically your use and feedback on the website of information resources and/or the interactions you had with the genetics contact service/teaching clinic [Note: wording will be selected as appropriate for the participant].

Before we start this interview, can I confirm that you have read the Information Statement that was provided when you were first invited to participate in this research? We sent it again with the email invitation. Do you have any questions about the interviews?

I am now going to switch on the digital recorder and record your consent. Is this ok? [Digital recorder will be turned on and participant will be asked]:

- Do you consent to participate in this interview?
- Do you consent to the interview being audio-recorded?

*Note for HREC: Sample questions below, to be asked as relevant to participants. The conversational style of the semi-structured interview will mean that the interviewer will allow the interviewee to freely comment on topics related to genomic testing and therefore the interview may not flow in the linear manner shown in the guide below. Additional questions and prompts, within the scope of these topics, will be asked in response to the interviewee’s comments.*

| **Broad topic** | **Example Questions** |
| --- | --- |
| Background | Specialty area. Years of practice. Previous experience with genomic testing [ask using the survey wording]  Probe: clinician characteristics (area of speciality, general interest in genomics, desire to learn more about genetics), practice characteristics (eligible patients, location) |
| Use of resource package/Website | How did you find out about the webpage? |
|  | Could you tell me about your use of the webpage? For example how frequently did you use the webpage? Which aspects of it did you use and why?  Probe: for yourself, with patients  How appropriately do you feel it was pitched? Did you have any challenges understanding any of the content?  If you used it with patients, how was that? What worked well? What didn’t work well?  Prompt: what would need to change in it, or what additional info/support/edu would you need to make use of its  Did your use of the package change over time / as you used it with more patients? |
|  | Thinking about the user-experience of this webpage, how was it for you?  Probe: what worked well? What didn’t work well? Any access issues? What could be improved about how you as a user interacted with this webpage?  What was useful in this webpage?  To what extent did the use of the resource package section help resolve the problems you were using it to address?  What wasn’t useful or was not as you had expected/What would you like to see but was missing? |
| Sustainability | If this webpage were to be made available on an ongoing basis such as on a public website not requiring specific access, what would need to change?  Where would you go to find something like this? (Where do you expect it to be hosted?)  How would we get the word out that this webpage is available? How would you recommend we advertise it to paediatricians?  Who should be involved in communicating and disseminating the webpage to paediatricians? |
| Use of genetics contact service | What prompted you to contact this expert?  [if they haven’t used it but are aware of it, what do you think would prompt you to use it? What do you think you would use it for – e.g. advice on consent, understanding test processes]  How often did you contact it? What for?  (advice on consent, discussing testing with patients, what information to provide when requesting the test, understanding test processes).  Did your use of the service change over time/with experience? |
|  | If you used it, how helpful did you find it?  Probe: in discussing the test with patients/parents, in understanding test ordering processes  To what extent do you think the service has helped resolve the problems that prompted you to contact it? |
| Sustainability | Do you feel there is a need for a service like this to be made available on an ongoing basis?  How might such a service be implemented? / How would you expect it to operate?  What else do you think a service like this might need to address in the future?  [e.g. if focussing at the moment on pre-test and test processes, future might need to provide support on result interpretation and follow up for families; or starting with advice for any patients but as confidence grows to manage straightforward requests, accessing this service for more complex patients] |
| Use of the teaching clinic | How did you hear about the clinic? What prompted you to book in to the teaching clinic?  How did you use the clinic?  What kind of consultations did you observe/ take part in?  (advice on consent, discussing testing with patients, what information to provide when requesting the test, understanding test processes)  As a learning opportunity, what worked well for you? What would you change or have preferred been done differently?  At the start of the clinic, you had some learning goals. What was important to you to learn from this clinic? [prompt if they don’t recall learning goals: genomics knowledge/skills, procedural knowledge/skills, observe how someone else practices]  Can you tell me about the new knowledge and/or skills you now have?  What gaps do you think you still have? Prompt: in knowledge and/or skills?  Did you participate in a consultation with one of your regular patients? If so, what do you think the positives and negatives of this were for both yourself and the family? |
| Sustainability | Do you feel there is a need for a service like this to be made available on an ongoing basis?  How might such a service be implemented? / How would you expect it to operate?  What else do you think a service like this might need to address in the future?  [e.g. if focussing at the moment on pre-test and test processes, future might need to provide support on result interpretation and follow up for families; or starting with advice for any patients but as confidence grows to manage straightforward requests, accessing this service for more complex patients] |
| Experience of testing | Have you noticed changes in your confidence about ordering genomic testing for patients? What do you think has contributed to these changes / or, why do you think your confidence hasn’t shifted even with access to these resources or services?  Have you ordered any tests for patients since using these interventions?  If yes, tell us about your experiences  Do you think you’d order again?  Please explain why/why not?  Thinking about your peers, do you know if they are using genomic testing in their practice? Is genomic testing a topic of conversation for paediatricians? What experiences have you heard about?  With these colleagues in mind, what do you think might be their response if they were provided with these same resources and service? |
| Final feedback | Thank you again for your time using these resources and contact service, and also taking part in the interview today.  Do you have any other comments or feedback you would like to give about this topic or the specific resources?  Is there anything I haven’t raised today that you were expecting to be asked about?  Thank you – wrap up. |

Genetic Expert Interview guide

Thank you for making the time for this research interview. We will be talking about your experiences with supporting paediatricians who are requesting genomic testing.

Before we start this interview, can I confirm that you have read the Information Statement that was provided to you? Do you have any questions about the interviews?

I am now going to switch on the digital recorder and record your consent. Is this ok?

Digital recorder will be turned on and participant will be asked:

- Do you consent to participate in this interview?
- Do you consent to the interview being audio-recorded?

*Note for HREC: Sample questions below, to be asked as relevant to participants. The conversational style of the semi-structured interview will mean that the interviewer will allow the interviewee to freely comment on topics related to genomic testing and therefore the interview may not flow in the linear manner shown in the guide below. Additional questions and prompts, within the scope of these topics, will be asked in response to the interviewee’s comments.*

| **Broad Topic** | **Example questions** |
| --- | --- |
| Introduction | What is your position in the genetics service/department? How long have you been working in that role?  What attracted you to the role in this project/service?  What did you think your role might be? How did you form that view? Was it what you were expecting? What was the same or different to what you were expecting?  What similarities or differences have you experienced when working in this project as compared to your current/previous role in the genetics service/department? |
| Use of resource package | As you know, in addition to the opportunity to contact a genetic expert, we have prepared an information resource for paediatricians. Did you use these resources with any of the paediatricians you were working with?  If so, in what way? If not, why? What did you do instead?  Did you recommend or direct paediatricians to the resource package? What prompted you to do that? Do you have a sense of what items might have been of most interest? Or greatest use? Is there anything you think could be added to that resource? Or removed? |
| Implementation | How did paediatricians make use of the genetic expertise contact service you offered?   - What did people ask you about (overall) - Were there any patterns in use over time? Did you get repeat calls from certain paediatricians and see any evolution over time (e.g. with the types of questions asked) - How would you describe the categories of cases that paediatricians were asking about? E.g. straightforward developmental delay? Complex family circumstances, children who could have had these investigations some time ago? - At what stage in the testing process were paediatricians contacting the service? E.g before talking to patients, after talking to patients to help with paperwork, after result return? What were your experiences of these different interactions?   What was your experience of doing this role?   - Did you have the resources and support you needed to do it? - Did the way you work evolve over time?   How do you perceive your skills/competencies in handling the calls/emails you received?  Were there calls you needed to refer on? What happened then?  Did you need to do follow up work after a call and then get back to the paediatrician? What sorts of things were you following up? |
| Diffusion of innovation | We are interested in how having access to someone like you via this service might impact on the attitudes, confidence and skills of paediatricians. Thinking about this, what is your sense of how the interactions you’ve had with paediatricians might have had these effects?  What else might be influencing paediatricians’ practice in this area? E.g. demand from patients, growing awareness of colleagues using genomics, improvements and changes to testing processes  What do you think is the next challenge for paediatricians to request funded genomic tests in their practice? E.g returning results to patients? Testing of other siblings? Re-analysis if no genetic cause reported? What might be strategies to address those challenges? What do you see as the role or interactions of genetic counsellors with paediatricians as genomic testing becomes more routine? |
| Close | Thank you for your time today and your responses to my questions. Was there anything else about this topic that you would like to raise or you thought I might ask you about?  Thank you again – wrap up |
