# Supplementary material for: Exomes in Paediatrics: Co‐Design and Implementation of Interventions to Support Paediatricians to Provide Genomic Care
Source: J Paediatr Child Health. 2025 Nov 20;62(1):97–105. doi: 10.1111/jpc.70237 (PMC12800879; doi:10.1111/jpc.70237)
Supplement: Supplementary file 5 — File S5: jpc70237‐sup‐0005‐FileS5.docx. [file JPC-62-97-s004.docx]

**Supporting Information File 5, qualitative quotes**

B. Dawson‐McClaren, M. Martyn, E. Weisz, et al., “Exomes in Paediatrics: Co‐Design and Implementation of Interventions to Support Paediatricians to Provide Genomic Care,” *Journal of Paediatrics and Child Health* (2025): 1–9, https://doi.org/10.1111/jpc.70237

Table 1: Representative quotes from evaluation interviews with paediatricians, mapped to the RE-AIM framework domains

| **Framework Domain** | **Representative Quotes** |
| --- | --- |
| REACH: At the individual level, did we reach our target population of general paediatricians with the interventions? | “you came to us, I think there was a teaching session at [name of service] and it was an online session. Yeah, which was just really good but I was on call so I had to patch in and out of the conversation, it's something really good and I was missing it but I took that link [to the website] and ran away with it” PAEDIATRICIAN 5  “I've been to a couple of talks, by [the study paediatrician], she came and gave a talk to us at our [name of service] practice and I listened to her talk at, I think the Royal Children's update [conference] last year” PAEDIATRICIAN 7  “I had referred a patient to genetics [department] and I think that’s how it happened, they said ‘do I want to come to the appointment’” PAEDIATRICIAN 9  “I went to a conference and they had a whole session on, it was sort of about disability, I think, and neurodevelopment and they had whole session on rare disorders and neurodegenerative disorders and then they actually spotlighted a few really good websites, and I think someone there had brought, had given it as an example” PAEDIATRICIAN 2 |
| EFFICACY/EFFECTIVENESS: What was the change (positive, negative, neutral) in paediatrician confidence and familiarity following intervention access? | “I found it [the website] really very helpful. There is a lot of information. So sometimes trying to work out exactly which bit of the information I need, but I mean took me very minimal time to work that out. But yeah, it's excellent. It's very well written the resources are all linked, understanding how to order the test, etcetera. It's everything is there… If I was to give it [my confidence now] a score out of 10, I would give it a 7 or an 8” PAEDAITRICIAN 4  I learned a couple of things. I think the first thing is that, well, my eyes aren’t very sharp and they don’t quite pick up the morphological differences and it was really good learning from, you know, watching [study paediatrician] do it [in the teaching clinic]. I don't usually compare all three family members directly and that's how I probably missed it and just because I look at the kid only usually or just with the one parent. So it was interesting to see that aspect of clinical photography and how that fits into it, from that perspective and it was also very reassuring to know that the whole exome sequence is usually going to pick up a lot of the conditions” PAEDIATRICIAN 5  I learned about really simplifying the outcomes and making sure that the patient is really aware of the four different outcome possibilities and understanding how they feel if they were to get one of the alternate outcomes… I feel more confident having done the clinic” PAEDIATRCIAN 8  “Specifically, the language around counselling and describing rather than you know, positive and negative describing the uncertainty… think it was just extremely useful to be able to actually see it happen and to have the opportunity to do some counselling with the supervision and feedback” PAEDIATRICIAN 10  “It was important for me to know their [parents] attitude towards the uncertain changes and how I should be going about those with families. You know, when I was going about explaining those the right way. That was the main thing because that’s my biggest concern with these tests…. I had a lot of questions about the variations of uncertain significance, I had questions about different labs and what they actually look for, I had some questions that sort of weren’t answered specifically which in a way was kind of helpful because like, as I said, it sort of helped me understand that there’s not always easy answers for some of these questions” PAEDIATRICIAN 9 |
| ADOPTION: Did paediatricians, and across what settings, use the intervention(s)? | “I have saved it [website] as a bookmark. See I have a lot of patients who I would be, this genetic evaluation would be really helpful,” PAEDAITRICIAN 1  “I got on the website, and I basically used the website really to learn as much as I could about it, so that then I could have a bit of a conversation with them [families]. I wanted to figure out how comfortable I felt in terms of my own knowledge and experience. [Now I] know a little bit more about what might be involved I could then either direct the parents in the right path or potentially support them in the process of actually doing it.” PAEDIATRICIAN 2  “I felt like I needed to take responsibility for my learning in that way, and I thought it would be really good to have a chat with the genetic counsellor to kind of unpack my clinical question and learn a bit more about the testing and then figure out where my gaps in knowledge were and then basically what I needed to do in order to support the family to take the next steps” PAEDIATRICIAN 2 |
| IMPLEMENTATION: Were the interventions used as intended? E.g what was used, how was it used and what wasn’t used? | “I did look at sort of the steps in terms of ordering the whole exome sequencing and then [study paediatrician] showed it to me again during the clinic. I think it's a really useful resource and probably what I will say I haven’t had a chance yet to order the whole exome sequence test. But if I like if it comes up again, that's the resource that I would go to in terms of guiding me step by step. And probably what I'd also recommend other people look at it, if they're thinking about ordering it.” PAEDIATRICIAN 11  “[I access the website] when I want to remind myself the [Medicare item] number. Previously I was looking at it because I didn't remember the age limit and all that, but I can now. And also I find that the patient part, the patient information. Like how, what, why do we do genetic testing like this?... plain English one that I use a lot” PAEDIATRICIAN 3  “One [key learning in the teaching clinic] was her dysmorphology examination, that's not something that I get to practice very often. As in the full dysmorphology examination, I do a lot of facial features, etcetera. And also the, I guess just the explanation to the family around just genetics in itself, she had some very useful phrases” PAEDIATRICIAN 4  “the practicalities of how to do it, I think was what came across really well in the videos…it helped dispel a lot of, I guess misconceptions or concerns” PAEDIATRICIAN 5 |
| MAINTENANCE: Has there been change in confidence and practice (test requests) over time? | “One of the things that I learned, sometimes it’s just practical tips. Like with dysmorphology, it’s easier to take photos and then look at them later on, rather than attempting to do it in the clinic. Which I think was a really great advice and I’ve implemented that into my practice routinely now” PAEDIATRICIAN 8  “I learnt a bit more about the online resources that are available to families around, which can be quite helpful, so I think that has kind of changed my practice since then in a way. So I’ve had one patient this week, one patient last week who I’ve given the information to and told them to go away and read it and let me know if they want to talk more” PAEDIATRICIAN 9  “I think it's [ordering funded genomic tests] something that I'll be doing in my day-to-day practise, going forward. It does, it’s very time intensive and I think just with more practise I will get better at doing that…You know, there's such long wait lists for families and being upskilled you're able to provide potentially more timely access to genetic [tests], where that's indicated” PAEDIATRICIAN 4 |
