# Supplementary material for: Exomes in Paediatrics: Co‐Design and Implementation of Interventions to Support Paediatricians to Provide Genomic Care
Source: J Paediatr Child Health. 2025 Nov 20;62(1):97–105. doi: 10.1111/jpc.70237 (PMC12800879; doi:10.1111/jpc.70237)
Supplement: Supplementary file 6 — File S6: jpc70237‐sup‐0006‐FileS6.docx. [file JPC-62-97-s005.docx]

**File S6, geographical distribution of test request patterns over study period**

B. Dawson‐McClaren, M. Martyn, E. Weisz, et al., “Exomes in Paediatrics: Co‐Design and Implementation of Interventions to Support Paediatricians to Provide Genomic Care,” *Journal of Paediatrics and Child Health* (2025): 1–9, https://doi.org/10.1111/jpc.70237


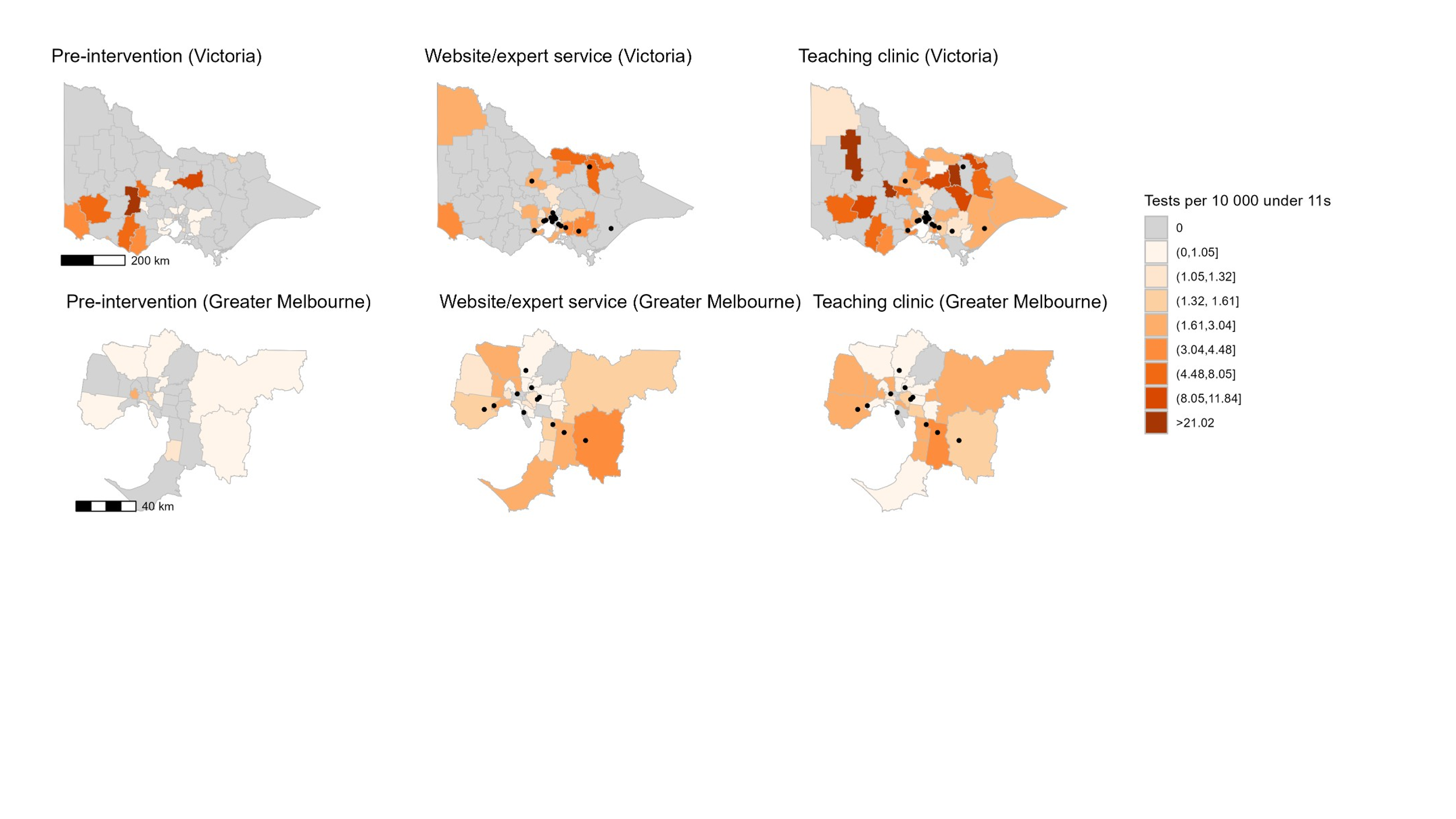


Figure S3: Tests ordered over the study time period, per 10 000 children aged under 11 years old.

The distribution of test requests across the State of Victoria was calculated by the number of tests per 10,000 children aged under 11 in each local government area (LGA). Laboratory data of conducted tests ordered by paediatricians are reported as those coded to the Medicare item numbers for childhood syndromes. Data from the Australian Bureau of Statistics (ABS) were used to obtain population estimates^[[1]](#endnote-2)^ and correspondences for postcodes to LGAs.^[[2]](#endnote-3)^

Data were mapped using R statistical software (v4.3.0),^[[3]](#endnote-4)^ the packages *sf*,^[[4]](#endnote-5)^ *dplyr,^[[5]](#endnote-6)^ ggplot2*,^[[6]](#endnote-7)^ *ggthemes*,^[[7]](#endnote-8)^ *classInt*,^[[8]](#endnote-9)^ *rgeoda*,^[[9]](#endnote-10)^ *ggspatial*,^[[10]](#endnote-11)^ *gridExtra*,^[[11]](#endnote-12)^ *cowplot*,^[[12]](#endnote-13)^ and shape files from the ABS.^[[13]](#endnote-14)^

Grey areas are areas where no tests were conducted, and darker colours indicate areas with higher rates of testing. Black dots indicate where awareness raising sessions were conducted in Victoria. Note, some LGAs have small numbers of children aged under 11, so standardised rates appear high.

Footnotes:

1. Australian Bureau of Statistics. General Community Profile DataPack– Victoria [Internet]. 2021 [cited 2025 Jan 23]. Available from: <https://www.abs.gov.au/census/find-census-data/datapacks> [↑](#endnote-ref-2)
2. Australian Bureau of Statistics. ASGS Geographic Correspondences (2021) Edition 3 [Internet]. 2021 [cited 2025 Jan 9]. Available from: <https://data.gov.au/data/dataset/asgs-edition-3-2021-correspondences> [↑](#endnote-ref-3)
3. R Core Team. R: A language and environment for statistical computing [Internet]. Vienna (Austria): R Foundation for Statistical Computing; 2021. Available from: [https://www.R-project.org/](https://www.r-project.org/) [↑](#endnote-ref-4)
4. Pebesma E. Simple Features for R: Standardized Support for Spatial Vector Data. R J. 2018;10(1):439–46. [↑](#endnote-ref-5)
5. Wickham H, François R, Henry L, Müller K, Vaughan D. dplyr: A Grammar of Data Manipulation [Internet]. Version 1.1.4. 2023 [cited 2025 May 5]. Available from: <https://github.com/tidyverse/dplyr>, [https://dplyr.tidyverse.org](https://dplyr.tidyverse.org/) [↑](#endnote-ref-6)
6. Wickham H. ggplot2: Elegant Graphics for Data Analysis. New York: Springer-Verlag; 2016. Available from: [https://ggplot2.tidyverse.org](https://ggplot2.tidyverse.org/) [↑](#endnote-ref-7)
7. Arnold J. ggthemes: Extra Themes, Scales and Geoms for 'ggplot2' [Internet]. Version 5.1.0.9000. 2024 [cited 2025 May 5]. Available from: <https://github.com/jrnold/ggthemes>, <https://jrnold.github.io/ggthemes/> [↑](#endnote-ref-8)
8. Bivand R. classInt: Choose Univariate Class Intervals [Internet]. Version 0.4-11. 2024 [cited 2025 May 5]. Available from: <https://github.com/r-spatial/classInt/>, <https://r-spatial.github.io/classInt/> [↑](#endnote-ref-9)
9. Li X, Anselin L. rgeoda: R Library for Spatial Data Analysis [Internet]. Version 0.1.0. 2025 [cited 2025 May 5]. Available from: <https://geodacenter.github.io/rgeoda/>, <https://github.com/geodacenter/rgeoda/> [↑](#endnote-ref-10)
10. Dunnington D. ggspatial: Spatial Data Framework for ggplot2 [Internet]. 2023 [cited 2025 May 5]. Available from: <https://paleolimbot.github.io/ggspatial/>, <https://github.com/paleolimbot/ggspatial> [↑](#endnote-ref-11)
11. Auguie B, Antonov A. gridExtra: Miscellaneous Functions for "Grid" Graphics [Internet]. Version 2.3. 2017 [cited 2025 May 5]. Available from: <https://cran.r-project.org/web/packages/gridExtra/index.html> [↑](#endnote-ref-12)
12. Wilke C. cowplot: Streamlined Plot Theme and Plot Annotations for 'ggplot2' [Internet]. Version 1.1.3. 2024 [cited 2025 May 5]. Available from: <https://wilkelab.org/cowplot/> [↑](#endnote-ref-13)
13. Australian Bureau of Statistics. Local Government Areas - 2021 - Shapefile [Internet]. 2021 [cited 2025 Jan 16]. Available from: <https://www.abs.gov.au/statistics/standards/australian-statistical-geography-standard-asgs-edition-3/jul2021-jun2026/access-and-downloads/digital-boundary-files> [↑](#endnote-ref-14)
